# ARL11 regulates lipopolysaccharide-stimulated macrophage activation by promoting mitogen-activated protein kinase (MAPK) signaling

**DOI:** 10.1074/jbc.RA117.000727

**Published:** 2018-04-04

**Authors:** Subhash B. Arya, Gaurav Kumar, Harmeet Kaur, Amandeep Kaur, Amit Tuli

**Affiliations:** From the Division of Cell Biology and Immunology, CSIR-Institute of Microbial Technology (IMTECH), Chandigarh 160036, India

**Keywords:** macrophage, lipopolysaccharide (LPS), GTPase, mitogen-activated protein kinase (MAPK), extracellular-signal-regulated kinase (ERK), ARL11, ARLTS1, Salmonella

## Abstract

ADP-ribosylation factor-like GTPase 11 (*ARL11*) is a cancer-predisposing gene that has remained functionally uncharacterized to date. In this study, we report that ARL11 is endogenously expressed in mouse and human macrophages and regulates their activation in response to lipopolysaccharide (LPS) stimulation. Accordingly, depletion of ARL11 impaired both LPS-stimulated pro-inflammatory cytokine production by macrophages and their ability to control intracellular replication of *Salmonella.* LPS-stimulated activation of extracellular signal–regulated kinase (ERK) and p38 mitogen-activated protein kinase (MAPK) was substantially compromised in *Arl11*-silenced macrophages. In contrast, increased expression of ARL11 led to constitutive ERK1/2 phosphorylation, resulting in macrophage exhaustion. Finally, we found that ARL11 forms a complex with phospho-ERK in macrophages within minutes of LPS stimulation. Taken together, our findings establish ARL11 as a novel regulator of ERK signaling in macrophages, required for macrophage activation and immune function.

## Introduction

*ARL11*, also known as ADP-ribosylation factor-like tumor suppressor gene 1 (*ARLTS1*), is a member of the Arf-like (ARL) family of small GTP-binding proteins that regulate diverse cellular processes, including vesicular trafficking, cytoskeletal organization, signaling, and ciliogenesis (1, 2). Similarly to the other members of the RAS superfamily of small GTPases, ARL proteins also function as molecular switches that cycle between inactive (GDP-bound) and active (GTP-bound) conformations. *ARL11* was first identified in a screening for putative tumor suppressor genes at chromosome location 13q14.3, a region frequently deleted in a variety of sporadic and hereditary hematopoietic and solid tumors (3–6). Subsequent studies reported down-regulation of *ARL11* expression in several sporadic lung cancer and ovarian tumors attributed to promoter methylation and loss of heterozygosity at the *ARL11* gene locus (7, 8). Further support for its tumor suppressor function has come from the finding that SNPs G446A (W149X) and T442C (C148R) in the *ARL11* gene are associated with familial risk for chronic lymphocytic leukemia (CLL)^6^ and for breast, prostate, and colorectal cancers (9–15). On the other hand, ectopic expression of ARL11 in lung carcinoma was reported to induce apoptosis, suggesting that ARL11 down-regulation promotes tumor cell survival (8).

A high degree of conservation of *ARL11* homologs in metazoans such as zebrafish, *Drosophila*, *Arabidopsis*, and mammals suggests an important cellular function of this gene. However, thus far, the physiological role of ARL11 is not known. Expression studies have revealed that mammalian *ARL11* transcripts are mostly abundant in lymphoid tissues (spleen, bone marrow, and lymph nodes), which is also supported by co-expression analysis from data mining approaches (8, 14).

We also searched for *Arl11* transcript expression in different immune cell types compiled in the Immunological Genome Project (ImmGen) database (https://www.immgen.org/)^7^ (37) and found that transcripts of *Arl11* predominated in macrophages, followed by monocytes and neutrophils. This led us to investigate the function of this uncharacterized protein in macrophages.

Here, we demonstrate that ARL11 expression is up-regulated upon lipopolysaccharide (LPS) stimulation in macrophages and regulates the pro-inflammatory macrophage effector functions. ARL11 was required for LPS- or pathogen-mediated activation of ERK1/2 and p38 mitogen-activated protein kinases (MAPKs). Notably, ERK1/2 colocalized with ARL11 at the cortical actin structures, and the two proteins interacted with each other, dependent upon ERK1/2 phosphorylation status. Taken together, our findings reveal that ARL11 regulates activation of the ERK1/2 MAPK signaling pathway in response to LPS stimulation and thereby regulates multiple pro-inflammatory effector functions of macrophages.

## Results

### ARL11 is expressed in macrophages, and its expression is enhanced upon LPS stimulation

**Figure 1.**
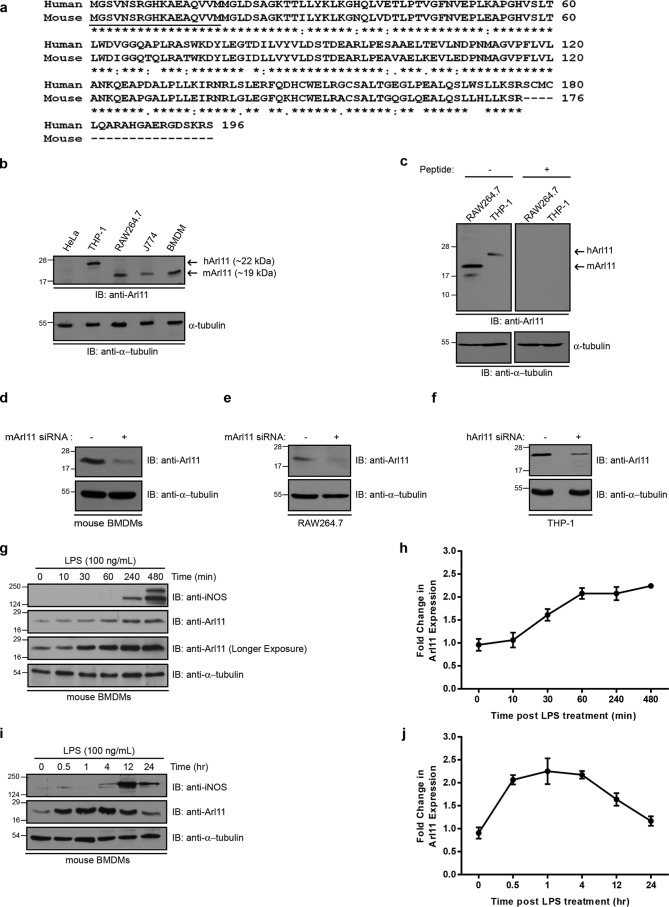
**ARL11 is endogenously expressed in macrophages of human and mouse origin, and LPS stimulation enhances ARL11 expression.** *a*, protein sequence alignment of human and mouse ARL11. The *underlined* sequence (identical between human and mouse ARL11 protein) represents the peptide sequence against which ARL11 antibody was generated. *b*, a representative Western blot (*IP*) depicting ARL11 protein expression in various human and mouse macrophage cell lines as well as in BMDMs. HeLa cell lysate was used as a negative control. *Arrows* indicate the specific band as labeled, and α-tubulin was used as the loading control. *c–f*, representative Western blots depicting the specificity of anti-ARL11 antibody using different approaches, including incubation of antibody with immunizing peptide that blocks the recognition of endogenous ARL11 band (*c*) and reduced signal of the endogenous ARL11 band upon siRNA transfection (*d–f*). *g–j*, BMDMs were treated with LPS for different time periods as indicated, and Western blot analysis of ARL11 was performed (*g* and *i*). Densitometric analysis of -fold change in ARL11 protein levels upon short-term (*h*) and long-term (*j*) LPS stimulation in BMDMs compared with untreated controls is plotted. Western blots for iNOS and α-tubulin were performed as a positive control for LPS treatment and to show an equal amount of protein loading, respectively. *Error bars*, S.D.

Computational analysis of *Arl11* transcript levels in different immune cell types using the ImmGen database revealed that *Arl11* was predominantly expressed in macrophages, monocytes, and neutrophils. To test this, we verified ARL11 expression in cell lysates from primary bone marrow–derived mouse macrophages (BMDMs), mouse macrophage cell lines (RAW264.7 and J774 cells), and a human monocyte-derived macrophage cell line (phorbol 12-myristate 13-acetate (PMA)-stimulated THP-1 cells) using an anti-peptide antibody raised against the N-terminal 17 amino acids of ARL11, a region that is identical in both human and mouse ARL11 protein (Fig. 1*a*; identical amino acids *underlined*). In accordance with a previous report, ARL11 expression was not observed in HeLa cell lysates (3). The antibody recognized bands at ∼19 and ∼22 kDa, respectively, corresponding to the theoretical molecular masses of mouse and human ARL11 protein (Fig. 1*b*). The antibody specificity in the Western blotting was confirmed by blocking with immunizing peptide and loss of signal observed in *Arl11* siRNA–transfected cell lysates (Fig. 1, *c–f*). Macrophage activation by engagement of cell-surface Toll-like receptors (TLRs) regulates expression of several proteins, which in turn are required for macrophage effector functions (16). To determine whether ARL11 expression is altered in activated macrophages, we performed a time-course analysis of LPS treatment on primary BMDMs. As shown in Fig. 1 (*g* and *h*), there was a ∼1.5-fold increase in the ARL11 protein level after 30 min of LPS treatment that further increased to ∼2-fold by 4 h. We observed similar increase in ARL11 protein level upon LPS treatment of RAW264.7 cells, a macrophage-derived cell line (Fig. S1, *a* and *b*).

Prior studies have shown that LPS treatment up-regulates marker of ER and oxidative stress in macrophages (17–19). We ruled out the possibility that ARL11 expression was induced as a result of ER or oxidative stress by treating primary BMDMs with thapsigargin and hydrogen peroxide, respectively (Fig. S1, *c* and *d*). We also noted that long-term/prolonged LPS stimulation of 12 and 24 h reduced ARL11 expression close to basal levels, suggesting that ARL11 expression is transiently up-regulated in activated macrophages (Fig. 1, *i* and *j*). The underlying regulatory mechanisms to keep ARL11 expression in check are probably required, as ARL11 is known to promote apoptosis (3, 6, 8). Indeed, overexpression of ARL11 induced apoptotic markers in primary BMDMs, as evident by the reduced levels of total poly(ADP-ribose) polymerase and increased caspase-3 cleavage (Fig. S1*e*). Of note, in primary BMDMs overexpressing ARL11, processing of the p19 N-terminal caspase-3 fragment containing the prodomain to the active p17 fragment was enhanced (20) (Fig. S1*e*, compare *lanes 1* and *2*). Further, ARL11 overexpression was also able to override the “delayed apoptosis” effect induced by LPS treatment of primary BMDMs lacking M-CSF signal (21, 22) (Fig. S1*e*, compare *lanes 7* and *8*). Taken together, these results demonstrate that ARL11 is expressed in human and mouse macrophages, and its expression is transiently up-regulated upon LPS stimulation.

### ARL11 is required for LPS-induced macrophage activation

**Figure 2.**
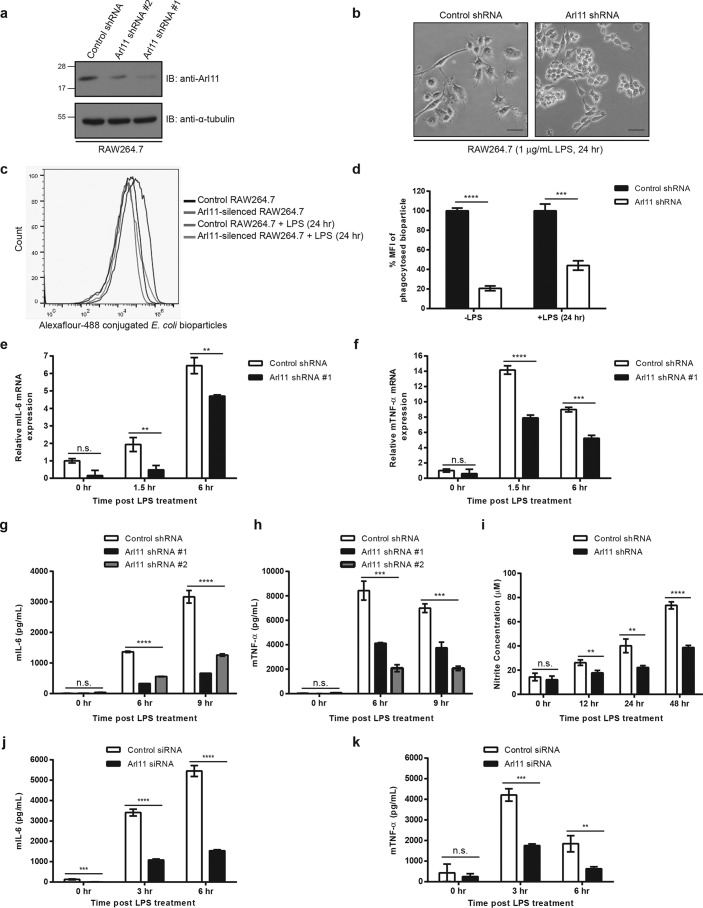
**Silencing of *Arl11* expression inhibits phagocytosis, LPS-induced pro-inflammatory cytokine secretion, and nitric oxide production in macrophages.** *a*, RAW264.7 cells were transfected with two different shRNAs (#*1* and #*2*) targeting *Arl11*, and the lysates from indicated treatments were immunoblotted (*IB*) with anti-ARL11 antibody to assess the knockdown efficiency and α-tubulin as the loading control. *b*, representative phase-contrast micrographs of control shRNA– and *Arl11* shRNA–transfected RAW264.7 cells stimulated with 1 μg/ml LPS for 24 h. A typical multiple-pseudopodia formation was observed in the case of control cells, whereas these morphological changes were inhibited upon *Arl11* silencing. *Bar*, 10 μm. *c* and *d*, phagocytosis of *E. coli* bioparticles in ARL11-depleted macrophages. Control shRNA– and *Arl11* shRNA–transfected RAW264.7 cells untreated or treated with 1 μg/ml LPS for 24 h were allowed to phagocytose Alexa Fluor 488–conjugated *E. coli* bioparticles. After 30 min of uptake, the cells were washed and analyzed by flow cytometry. The histograms show the flow cytometry results of analyzing the macrophages for Alexa Fluor 488 signal (*c*), and the percentage of mean fluorescent intensity (*MFI*) for Alexa Fluor 488 signal by the cells is plotted (*d*). Data shown represent mean ± S.D. (*error bars*) (*n* = 3; ***, *p* < 0.001; ****, *p* < 0.0001; Student's *t* test). *e–h*, production of pro-inflammatory mediators (IL-6 and TNFα) by *Arl11*-silenced RAW264.7 cells after LPS treatment was measured by quantitative RT-PCR (*e* and *f*) and ELISA (*g* and *h*). *i*, effect of ARL11 depletion on nitric oxide production. Control or *Arl11*-silenced RAW264.7 cells were treated with LPS, and nitrite production (an indication of the presence of nitric oxide) was evaluated in culture supernatants by the Griess reaction. *j* and *k*, BMDMs were transfected with control or *Arl11* siRNA. After 72 h of siRNA transfection, cells were stimulated with 100 ng/ml LPS for the indicated time periods, supernatants from the cultures were collected, and the concentration of IL-6 (*j*) and TNFα (*k*) was measured by ELISA. Data shown represent mean ± S.D. (*n* = 3) (*n.s.*, not significant; **, *p* < 0.01; ***, *p* < 0.001; ****, *p* < 0.0001; Student's *t* test).

To determine ARL11 function in macrophages, we next investigated whether its depletion affects macrophage effector functions. As shown in Fig. 2*a*, ARL11 expression was significantly reduced (>80%) in RAW264.7 cells treated with two different shRNA sequences targeted against mouse *Arl11*. To evaluate whether *Arl11* gene silencing alters cell proliferation, we determined the growth rate of control shRNA– and *Arl11* shRNA–transfected RAW264.7 cells using alamarBlue® dye reduction. No significant differences in the proliferation rate of ARL11-depleted RAW264.7 cells were observed as compared with the control cells (Fig. S2*a*). Next, we stimulated both control and *Arl11*-silenced RAW264.7 macrophages with LPS for 24 h and assessed the activation phenotype of these cells. Whereas typical pseudopodia formation was observed in LPS-stimulated control cells (morphology associated with macrophage maturation/activation), these changes in macrophage morphology were strongly suppressed upon ARL11 depletion (Fig. 2*b*, compare *left panel* with *right panel*).

To determine ARL11's role in regulating macrophage effector functions, we first examined the phagocytic ability of control and ARL11-depleted cells. To this end, we analyzed phagocytosis of Alexa Fluor 488–conjugated *E. coli* bioparticles in control and ARL11-depleted cells by flow cytometry. Notably, the phagocytic capacity of *Arl11*-silenced macrophages was significantly lower (∼2.5-fold) compared with control cells under both normal and LPS-stimulated conditions (Fig. 2*c*; quantification shown in *d*). Next, we determined pro-inflammatory cytokine production in control and ARL11-depleted RAW264.7 cells upon LPS stimulation. *Arl11* silencing led to significantly lower IL-6 and TNFα production, as determined by quantitative RT-PCR and enzyme-linked immunoassay (Fig. 2, *e–h*). In addition to pro-inflammatory cytokines, nitric oxide production (measured as nitrite concentration) was significantly lower in ARL11-depleted cells as compared with control (Fig. 2*i*; control shRNA, ∼4.6-fold; *Arl11* shRNA, ∼3-fold increase from 0 to 48 h). We noted that the impaired LPS-mediated effector responses in ARL11-depleted RAW264.7 cells were not due to reduced cell-surface levels of TLR4, suggesting that ARL11 acts downstream of the TLR4 pathway (Fig. S2*b*). We corroborated these findings in primary BMDMs and PMA-stimulated THP-1 macrophages. As in RAW264.7 macrophages, pseudopodia formation was suppressed in *ARL11*-silenced THP-1 macrophages upon LPS stimulation (Fig. S3, *a* and *b*). Further, levels of pro-inflammatory cytokine (IL-6 and TNFα) production were significantly reduced upon ARL11 depletion in both primary BMDMs and THP-1 macrophages (Fig. 2 (*j* and *k*) and Fig. S3 (*c* and *d*)). Taken together, these results suggest that LPS induces ARL11 expression to activate downstream pro-inflammatory functions of macrophages.

### ARL11 positively regulates LPS-induced ERK1/2 activation in macrophages

**Figure 3.**
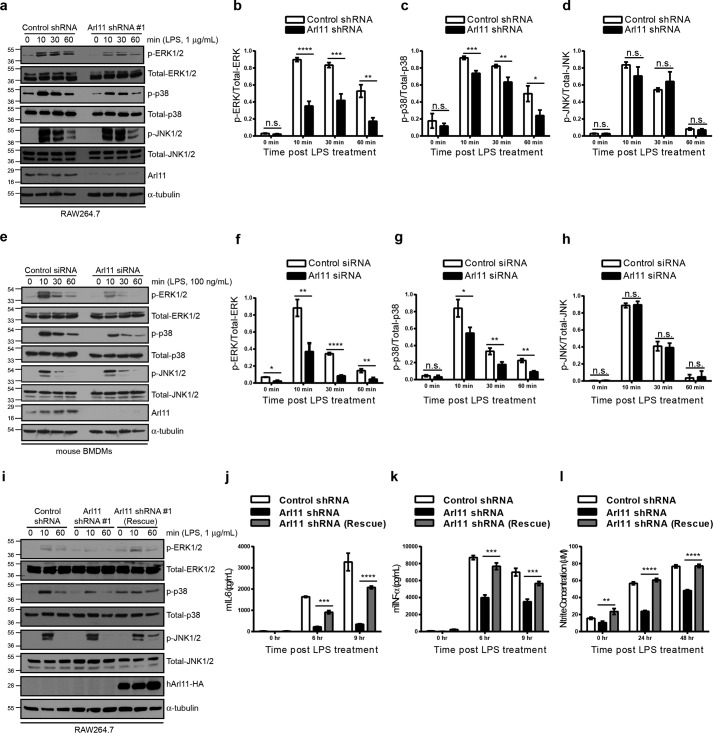
**ARL11 depletion impairs ERK1/2 and p38 MAPK phosphorylation in LPS-stimulated macrophages.** *a*, control shRNA– and *Arl11* shRNA 1–transfected RAW264.7 cells were treated with 1 μg/ml LPS for different time periods, and lysates were prepared and blotted with the indicated anti-phospho-antibodies. Total ERK1/2, p38, JNK1/2, and α-tubulin were probed as quantitative controls. *b–d*, densitometric analysis was performed to determine the ratio of phospho-ERK to total ERK (*b*), phospho-p38 to total p38 (*c*), and phospho-JNK to total JNK (*d*) in control shRNA– and *Arl11* shRNA 1–transfected RAW264.7 cells treated with 1 μg/ml LPS for different time periods, as indicated. *e*, BMDMs were transfected with control or *Arl11* siRNA. After 72 h of siRNA transfections, cells were stimulated with 100 ng/ml LPS for the indicated time periods, and the lysates were prepared and blotted with the indicated anti-phospho-antibodies. Total ERK1/2, p38, JNK1/2, and α-tubulin were probed as quantitative controls. *f–h*, densitometric analysis was performed to determine the ratio of phospho-ERK to total ERK (*f*), phospho-p38 to total p38 (*g*), and phospho-JNK to total JNK (*h*) in control siRNA– and *Arl11* siRNA–transfected BMDMs treated with 100 ng/ml LPS for different time periods as indicated. *i*, cell lysates of control shRNA–, *Arl11* shRNA–, and *Arl11* shRNA (rescue)–RAW264.7 cells were treated with LPS, and the lysates were prepared and blotted with the indicated anti-phospho-antibodies. Total ERK1/2, p38, JNK1/2, and α-tubulin were probed as quantitative controls. To confirm the expression of human *ARL11*-HA rescue plasmid, the lysates were probed with anti-HA antibody. *j–l*, control shRNA–, *Arl11* shRNA–, and *Arl11* shRNA (rescue)–RAW264.7 cells were treated with LPS for the indicated time periods, supernatants from the cultures were collected, the concentration of IL-6 (*j*) and TNFα (*k*) was measured by ELISA, and nitrite production (an indication of the presence of nitric oxide) was evaluated by the Griess reaction (*l*). Data shown represent mean ± S.D. (*error bars*) (*n* = 3) (*n.s.*, not significant; *, *p* < 0.05; **, *p* < 0.01; ***, *p* < 0.001; ****, *p* < 0.0001; Student's *t* test).

MAPKs (namely classical MAPK or ERK1/2, p38 kinase, and c-Jun N-terminal kinase (JNK1/2)) form the major signaling cascade that responds to LPS stimulation and promotes pro-inflammatory effector functions in macrophages (23, 24). To understand the mechanism of ARL11-regulated macrophage activation, we analyzed phosphorylation of MAPKs in both control and *Arl11*-silenced RAW264.7 cells and primary BMDMs stimulated with LPS for varying lengths of time. Notably, we found that phosphorylation of both ERK1/2 and p38 MAPKs was significantly lower in LPS-stimulated *Arl11*-silenced RAW264.7 cells and BMDMs, as compared with their respective controls, whereas the total levels of these kinases remained unchanged (Fig. 3, *a–h*). On the other hand, phosphorylation of JNK1/2 was not altered in ARL11-depleted macrophages (Fig. 3, *a* and *e*; quantification shown in *d* and *h*). Consistent with our observations, phosphorylation of p90RSK (90-kDa ribosomal S6 kinase), a downstream substrate of ERK1/2 kinase, was also reduced upon ARL11 depletion (Fig. S4, *a* and *e* (*top panels*); quantification shown in *b* and *f*). Similar results were obtained with a second shRNA sequence targeting *Arl11* in RAW264.7 cells and upon *ARL11* silencing in PMA-differentiated THP-1 cells (Fig. S3, *e* and *f*). Notably, levels of phospho-MEK1/2 and -MKK3/6, upstream mitogen-activated protein kinase kinases (MAPKKs) that activate ERK1/2 and p38, respectively, remained unchanged in ARL11-depleted macrophages, suggesting that ARL11 acts downstream of the MAPKK in the MAPK signaling pathway (Fig. S4, *a* and *e*; quantification shown in *c*, *d*, *g*, and *h*).

To confirm the specificity of our RNAi approach, we rescued the ARL11-depleted RAW264.7 cells by reintroducing the human ortholog that shares ∼86% identity in its amino acid sequence with mouse ARL11 (Fig. 1*a*). As shown in Fig. 3*i*, transfection of the human ortholog in ARL11-depleted RAW264.7 cells rescued LPS-induced ERK1/2 and p38 activation. In agreement with previous results, no differences in the phosphorylation status of JNK1/2 were observed among control shRNA–, *Arl11* shRNA–, and *Arl11* shRNA (rescue)–RAW264.7 cells. Notably, human ARL11 also rescued the LPS-induced pro-inflammatory cytokine (IL-6 and TNFα) and NO production in ARL11-depleted cells to a level similar to control (Fig. 3, *j–l*). Taken together, our findings reveal that ARL11 is required for LPS-induced ERK1/2 and p38 activation, which in turn promotes macrophage effector functions.

### ARL11 controls replication of Salmonella typhimurium inside macrophages

**Figure 4.**
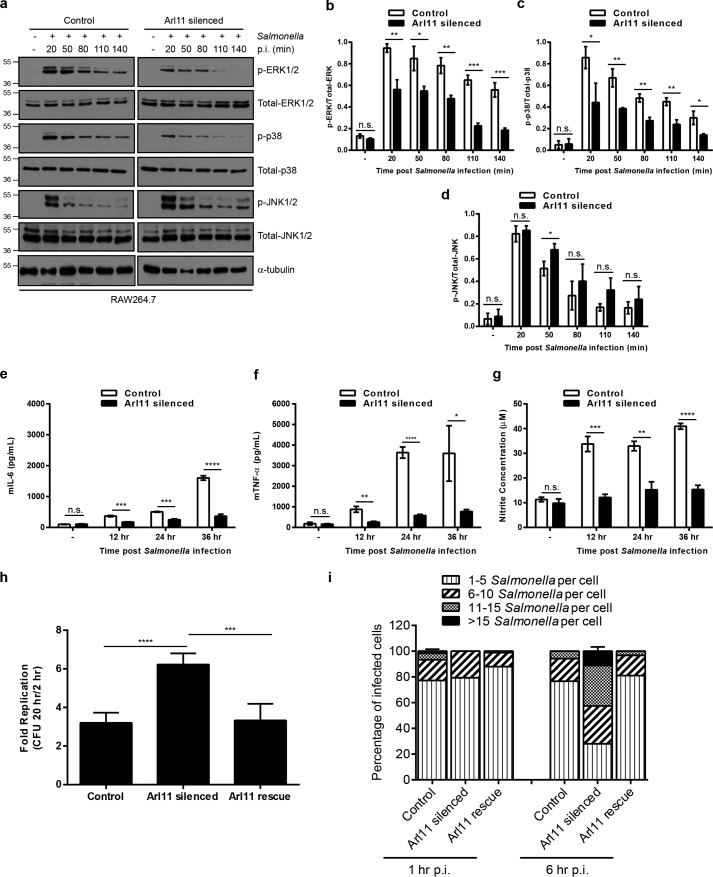
**ARL11 depletion in macrophages results in defective killing of intracellular *Salmonella*.** *a*, control and *Arl11*-silenced RAW264.7 cells were infected with *Salmonella* for different time periods, and lysates were prepared and blotted with the indicated anti-phospho-antibodies. Total ERK1/2, p38, JNK1/2, and α-tubulin were probed as quantitative controls. *b–d*, densitometric analysis was performed to determine the ratio of phospho-ERK to total ERK (*b*), phospho-p38 to total p38 (*c*), and phospho-JNK to total JNK (*d*) in control and *Arl11*-silenced RAW264.7 cells infected with *Salmonella* for different time periods, as indicated. *e–g*, control and *Arl11*-silenced RAW264.7 cells were infected with *Salmonella* for different time periods, supernatants from the cultures were collected, the concentration of IL-6 (*e*) and TNFα (*f*) was measured by ELISA, and nitrite production (an indication of the presence of nitric oxide) was evaluated by the Griess reaction (*g*). *h* and *i*, control, *Arl11*-silenced, and *Arl11* rescue RAW264.7 cells were infected with *Salmonella*, and the -fold change in recoverable cfu was calculated (20 h/2 h p.i.) by a gentamicin protection assay. By using confocal microscopy, the intracellular bacteria were counted in ∼100 cells/experiment. These numbers were grouped according to the *key* and expressed as a percentage of the total infected cell population (*i*). Data shown represent mean ± S.D. (*error bars*) (*n* = 3) (*n.s.*, not significant; *, *p* < 0.05; **, *p* < 0.01; ***, *p* < 0.001; ****, *p* < 0.0001; Student's *t* test).

We next investigated whether ARL11 is required for macrophage activation upon infection with live bacteria and for subsequent bacterial clearance by activated macrophages. To determine this, we infected control and *Arl11*-silenced RAW264.7 macrophages with *Salmonella typhimurium* for different time periods. Unlike the *Salmonella*-infected control shRNA–treated cells, where transient but substantial phosphorylation of ERK1/2 and p38 MAPKs was observed, ARL11-depleted cells had impaired MAPK activation in response to *Salmonella* infection (Fig. 4*a*; quantification shown in *b* and *c*). These findings suggest that ARL11 is required for pathogen-induced activation of ERK1/2 and p38 MAPKs in macrophages. Of note, a modest and transient increase in the phosphorylation of JNK1/2 was observed in *Salmonella*-infected *Arl11*-silenced RAW264.7 cells at initial time points when compared with control cells; however, it was decreased to the same level at later time points of *Salmonella* infection (Fig. 4*a*; quantification shown in *d*). Consistent with an impaired MAPK activation, pro-inflammatory cytokine (IL-6 and TNFα) and nitric oxide production was also significantly lower in *Salmonella*-infected *Arl11*-silenced macrophages (Fig. 4, *e–g*).

Because MAPK signaling underlies the immune responses of macrophages against intracellular pathogens (as evident by the ∼3.5-fold increased *Salmonella* load in macrophages treated with MEK inhibitor, U0126; Fig. S5*a*), we next investigated the role of ARL11 in regulating *Salmonella* replication inside macrophages. To this end, control shRNA- and *Arl11* shRNA-treated RAW264.7 cells were infected with *S. typhimurium*, and the standard cfu assay was performed to examine the intracellular bacterial load at 2 h and 20 h post-infection (p.i.). Consistent with an impaired macrophage effector function, a ∼2-fold increase in intracellular bacterial load was recovered in ARL11-depleted cells compared with control cells (Fig. 4*h*). These results were further corroborated by reintroduction of the human ortholog, which rescued the antibacterial activity of ARL11-depleted RAW264.7 macrophages (Fig. 4*h*). We independently confirmed these results by enumerating bacterial load using immunofluorescence microscopy of GFP-expressing *Salmonella*-infected macrophages at 1 and 6 h p.i. Consistent with a lower phagocytic index in ARL11-depleted cells (Fig. 2, *c* and *d*), we did not observe cells with 11–15 bacteria phagocytosed/cell upon *Arl11* silencing at 1 h p.i. At 6 h p.i., where ∼75% of control shRNA–treated cells had <5 bacteria/cell and ∼18% had 6–10 bacteria/cell, ARL11 depletion led to increased load, with ∼50% of cells having >10 bacteria/cell and ∼11% cells having >15 bacteria/cell. This defect in bacterial clearance in ARL11-depleted RAW264.7 macrophages was rescued upon reintroduction of the human ortholog (Fig. 4*i*; representative images shown in Fig. S5*b*). Overall, these findings suggest that ARL11 is a crucial component of the cellular host defense system required to limit intracellular bacterial replication.

### Increased ARL11 expression leads to exhaustion of LPS-stimulated macrophages

**Figure 5.**
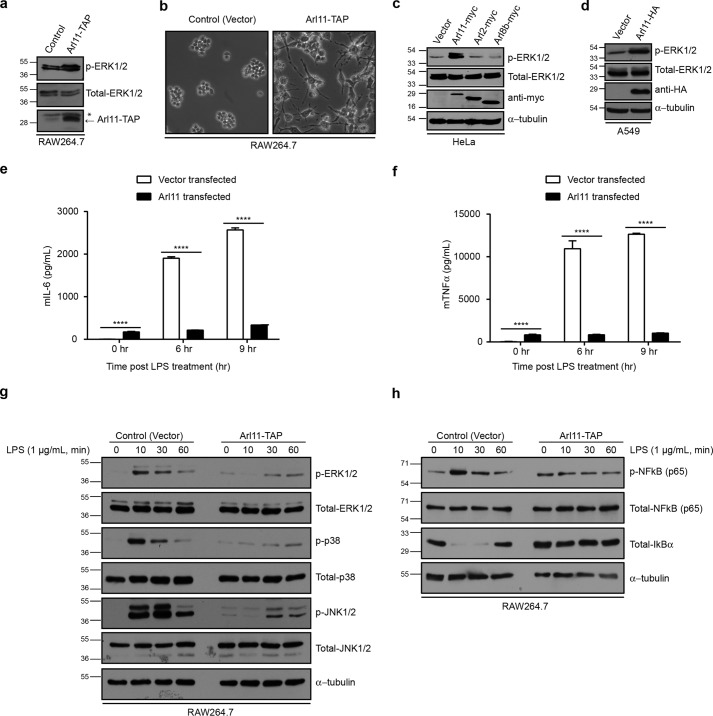
**ARL11 overexpression in macrophages was sufficient for ERK1/2 phosphorylation and impaired further stimulation of macrophages upon LPS stimulation.** *a*, RAW264.7 cells were transfected with plasmid encoding ARL11-TAP or empty vector. After 16 h, cells were lysed, and lysates were prepared and blotted with the indicated antibodies. The overexpression of ARL11-TAP was confirmed by probing with anti-TAP antibody. *, nonspecific protein band. *b*, representative phase-contrast micrographs of control and ARL11-TAP–overexpressing RAW264.7 cells. Overexpression of ARL11 leads to multiple-pseudopodia formation, a hallmark of activated macrophages. *Bar*, 10 μm. *c* and *d*, HeLa (*c*) and A549 (*d*) cells were transfected with the indicated ARL-encoding plasmids or empty vector. After 16 h, cells were lysed, and lysates were prepared and blotted with the indicated antibodies. *e* and *f*, overexpression of ARL11 inhibited LPS-induced production of pro-inflammatory cytokines. RAW264.7 cells expressing ARL11-TAP or empty vector–transfected were stimulated with 1 μg/ml LPS for the indicated time periods. Supernatants from the cultures were collected, and the concentration of IL-6 (*e*) and TNFα (*f*) was evaluated by ELISA. Data shown represent mean ± S.D. (*error bars*) (*n* = 3) (****, *p* < 0.0001; Student's *t* test). *g* and *h*, control and ARL11-TAP–overexpressing RAW264.7 cells were treated with 1 μg/ml LPS for the indicated time periods. Cell lysates were prepared and blotted with the indicated anti-phospho-antibodies. Total ERK1/2, p38, JNK1/2, NF-κB (p65), IκBα, and α-tubulin were probed as quantitative controls.

Having determined that *Arl11* silencing impairs macrophage effector functions in response to LPS stimulation, we turned to ask whether ARL11 is sufficient to activate the pro-inflammatory pathway in macrophages. Notably, RAW264.7 cells stably overexpressing *Arl11* (ARL11-TAP) had multiple pseudopodia, an effect generally observed in activated macrophages (Fig. 5 (*a* and *b*), compare *left panel* with *right panel*). Consistent with this observation, ARL11-TAP cells had higher levels of phosphorylated ERK1/2 at steady-state conditions as compared with control (vector-transfected) (Fig. 5*a*). Indeed, in a heterologous cell system, such as HeLa and A549 cell lines (where no endogenous ARL11 is present), ectopic ARL11 expression was sufficient to result in enhanced ERK1/2 phosphorylation (Fig. 5, *c* and *d*). This effect was limited to ARL11, as expression of other Arl family members, such as ARL2 and ARL8b, did not affect the phosphorylation status of ERK1/2 in these cell lines (Fig. 5*c*). These results strongly support a direct role of ARL11 in promoting and/or stabilizing MAPK activation.

We next evaluated macrophage effector functions in both control (vector)- and ARL11-TAP RAW264.7 cells upon LPS stimulation. The cell-surface levels of TLR4 in ARL11-TAP were similar to control, indicating that LPS recognition is not compromised upon ARL11 overexpression (Fig. S5*c*). Next, we examined pro-inflammatory cytokine (IL-6 and TNFα) levels in LPS-stimulated control and ARL11-TAP–transfected RAW264.7 cells. Surprisingly, ARL11-TAP cells failed to respond to LPS stimulation with little or no significant change in both IL-6 and TNFα production over time, as compared with control (vector)-transfected cells, where, expectedly, cytokine production increased with time (Fig. 5, *e* and *f*). Consistent with these observations, LPS stimulation failed to induce MAPK activation in ARL11-TAP cells, although these cells showed a higher MAPK phosphorylation under steady-state conditions (Fig. 5*g*). This failure to respond to LPS treatment was especially apparent after 10 min, when MAPK phosphorylation was suppressed even below the basal levels in ARL11-TAP cells and only weak activation was observed after 30 min of LPS treatment (Fig. 5*g*). LPS also stimulates NF-κB activation in macrophages, which in turn depends upon the activity of MAPKs (25–27). To evaluate NF-κB activation in LPS-stimulated ARL11-TAP cells, we monitored the phosphorylation of transactivation domain (serine 536) of p65 subunit of NF-κB (28). Indeed, LPS treatment rapidly induced p65 phosphorylation in vector-transfected but not in *Arl11*-TAP–transfected RAW264.7 cells (Fig. 5*h*). Notably, *Arl11*-TAP–transfected cells showed a significant p65 phosphorylation in unstimulated cells, consistent with their activated morphology (Fig. 5*b*). We also examined the degradation of IκBα (an inhibitor of NF-κB signaling) upon LPS stimulation, which was significantly less in *Arl11*-TAP–transfected cells compared with control (Fig. 5*h*, *third panel*). These results suggest that unregulated and higher expression levels of ARL11 in macrophages trigger tolerance toward LPS treatment, possibly due to negative feedback regulation of the MAPK signaling pathway.

### ARL11 exhibits nucleo-cytoplasmic distribution and interacts with phospho-ERK

**Figure 6.**
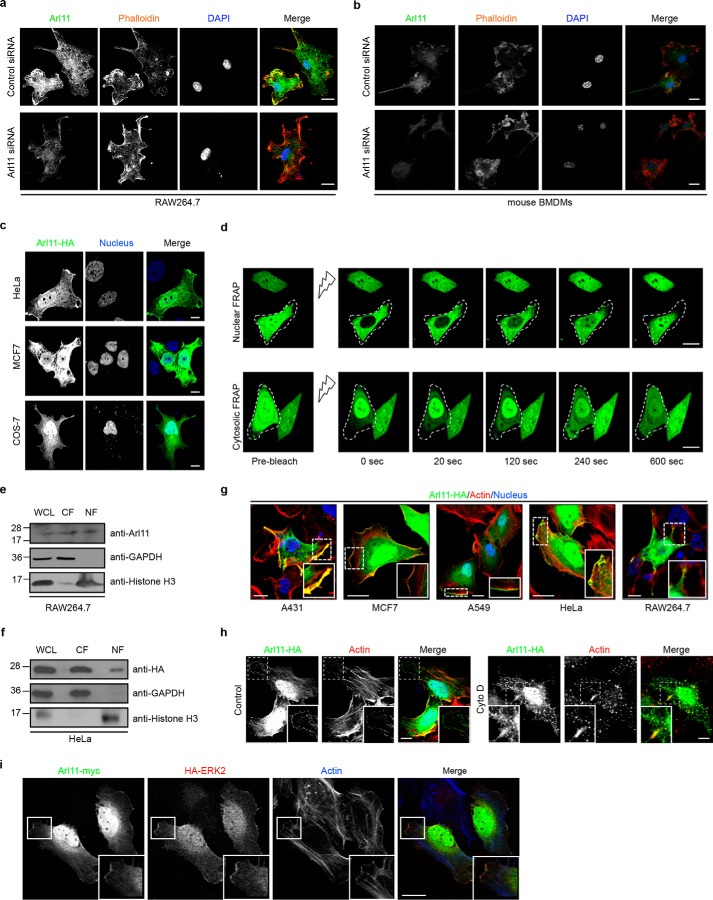
**ARL11 exhibits nucleo-cytosolic distribution and colocalizes with ERK on cortical actin structures.** *a* and *b*, representative confocal micrographs of RAW264.7 cells (*a*) and BMDMs (*b*) treated with either control siRNA or *Arl11* siRNA. After 48 h of siRNA treatment, cells were fixed and immunostained with anti-ARL11 antibody (*green*) to visualize endogenous staining of ARL11, actin was stained using phalloidin (*red*), and the nucleus was stained using DAPI. In *Arl11* siRNA–treated cells, the endogenous staining of ARL11 was not observed. *c*, representative confocal micrographs of HeLa, MCF7, and COS-7 cells transfected with *ARL11*-HA–expressing plasmid. Cells were fixed and immunostained with anti-HA antibody (*green*) to visualize *ARL11*-transfected cells, and the nucleus was stained using DAPI. *d*, nuclear and cytoplasmic FRAP analysis on HeLa cells transfected with ARL11-GFP plasmid. HeLa-ARL11-GFP cells were seeded into 35-mm live-cell imaging dishes, and photobleaching was performed by the 488-nm laser line at 100% intensity for 20 s at an ROI (∼5 μm^2^) near either the center of the nucleus (for nuclear FRAP) or in the cytoplasm (for cytoplasmic FRAP). Without any time-lapse post-bleach, fluorescence recovery was measured every 20 s for up to 10 min until the fluorescence recovery reached the plateau stage. Images acquired were processed in ImageJ software, and FRAP quantification is shown in Fig. S6 (*a* and *b*). *e* and *f*, lysates of RAW264.7 cells (*e*) or HeLa cells transfected with *ARL11*-HA plasmid (*f*) were subjected to nuclear/cytoplasmic fractionation. The isolated whole-cell lysates (*WCL*), nuclear fractions (*NF*), and cytosolic fractions (*CF*) were probed with anti-ARL11 antibody (for endogenous ARL11 distribution) in the case of RAW264.7 cells or anti-HA antibody (for ectopically expressed ARL11-HA) in the case of HeLa cells. The fractions were probed with anti-GAPDH and anti-histone H3 antibodies for checking the purity of cytosolic and nuclear fractions, respectively. *g*, ARL11 colocalized with the actin filaments in the lamellar ruffles. Representative confocal micrographs of the indicated cell type transfected with *ARL11*-HA plasmid are shown. After 12 h post-transfection, cells were fixed and immunostained with anti-HA antibody (*green*) to identify *ARL11*-transfected cells, actin was stained using phalloidin (*red*), and the nucleus was stained using DAPI. As shown in the *magnified insets*, ARL11 colocalizes with cortical actin. *h*, HeLa cells were transfected with *ARL11*-HA plasmid. After 12 h, cells were either left untreated (control) or treated with 5 μm cytochalasin D for 2 min to cause partial disruption of the actin cytoskeleton. Following treatment, cells were fixed and stained with anti-HA antibody (*green*) to visualize *ARL11*-transfected cells, actin was stained using phalloidin (*red*), and the nucleus was stained using DAPI. As shown in the *magnified insets*, the ARL11 pattern closely mirrored the drug-induced actin distribution. *i*, HeLa cells were cotransfected with *ARL11*-Myc– and HA-ERK2–expressing plasmids. After 12 h, cells were fixed and stained with anti-Myc antibody (*green*) to identify *ARL11*-transfected cells and anti-HA antibody (*red*) to visualize ERK2 signal, and actin was stained using phalloidin (*blue*). As shown in the *magnified insets*, ARL11 colocalizes with ERK at cortical actin. *Bars*, 10 μm (*main figure*) and 2 μm (*insets*).

We next investigated ARL11 localization in macrophages using antibody against the endogenous protein as well as by expressing an epitope-tagged construct of *ARL11* (ARL11-HA) in several mammalian cell lines, including HeLa, MCF7, and COS-7, cells. Notably, both endogenous ARL11 and ARL11-HA showed a similar nucleo-cytoplasmic distribution (Fig. 6, *a–c*). Consistent with immunofluorescence results, an ARL11 protein band was detected in the nuclear and cytosolic protein fractions in RAW264.7 macrophages as well as in HeLa cells transfected with ARL11-HA–expressing plasmid (Fig. 6, *e* and *f*). A similar distribution of ARL11 was also observed when expressed in additional cell lines (A431 and A549) or with a different tag (GFP) at either the N or C terminus (Fig. 6, *d* and *g*).

Next, we checked whether ARL11 undergoes nucleo-cytoplasmic shuttling. To this end, nuclear and cytosolic fluorescence recovery after photobleaching (FRAP) analysis was carried out with living cells that were transfected earlier with a plasmid expressing ARL11-GFP. For nuclear FRAP, we applied a confocal microscopy–based method to bleach the ARL11-GFP fluorescence inside the nucleus, and then fluorescence recovery was monitored over time. A loss in fluorescence in another, nonbleached compartment (*e.g.* the cytoplasm) means a dynamic shuttling of fluorescent molecules between the bleached and the nonbleached regions. Representative images from a typical FRAP experiment are presented in Fig. 6*d* (*top panel*; graph shown in Fig. S6*a*), where it can be observed that the ARL11-GFP signal in the nucleus in the photobleached cell recovers with time, whereas cytoplasmic ARL11-GFP fluorescence decreases steadily (Video S1). Similar movement of ARL11-GFP from the nucleus to the cytoplasm was seen when an area in the cytoplasm was photobleached (Fig. 6*d*, *bottom panel*; graph shown in Fig. S6*b*) (Video S2). Although our attempt at identifying a nuclear localization signal in ARL11 was unsuccessful, ARL11 export to the cytosol was nevertheless blocked upon depletion of the general nuclear export factor, CRM1 (chromosome region maintenance 1) (Fig. S6, *c–e*). These data suggest that ARL11 exhibits both nuclear and cytosolic distribution and dynamically shuttles between these two compartments.

**Figure 7.**
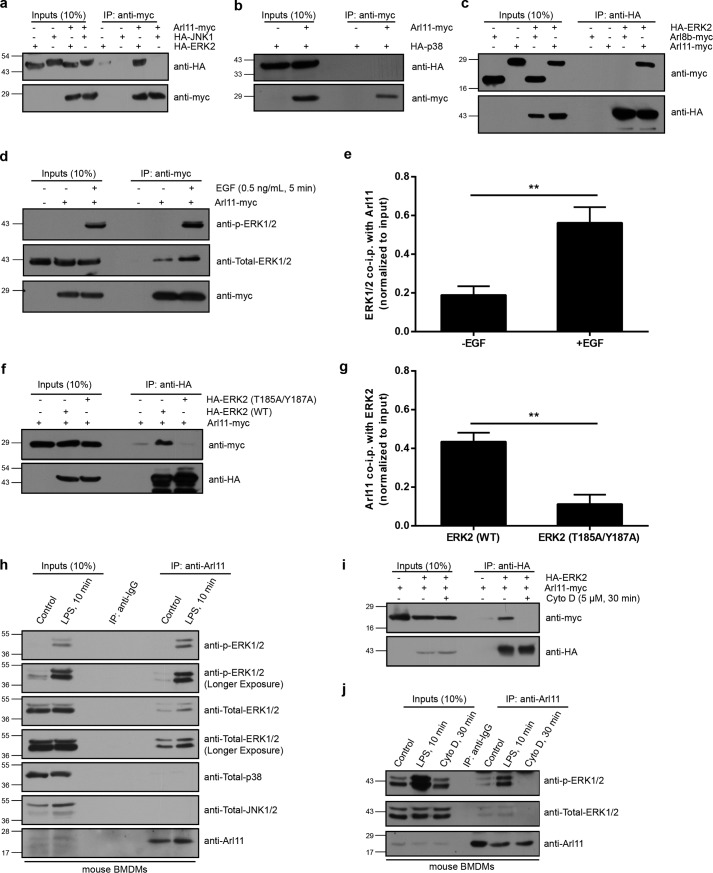
**ARL11 specifically interacts with the phosphorylated form of ERK.** *a*, HEK293T cell lysates expressing HA-ERK2 or HA-JNK1 alone or co-expressed with ARL11-Myc were immunoprecipitated (*IP*) with anti-Myc antibody resin, and the precipitates were immunoblotted using the indicated antibodies. *b*, lysates of HEK293T cells expressing ARL11-Myc alone or coexpressing ARL11-Myc and HA-p38 were subjected to immunoprecipitation with anti-Myc antibody resin, and the precipitates were immunoblotted with the indicated antibodies. *c*, ARL11 but not ARL8b interacts with ERK2. HA-ERK2 was cotransfected with *ARL11*-Myc or *ARL8b*-Myc into HEK293T cells; lysates were immunoprecipitated with anti-HA antibody resin, and the precipitates were immunoblotted with the indicated antibodies. *d* and *e*, HEK293T cells transfected with *ARL11*-Myc were either left untreated or treated with EGF (0.5 ng/ml) for 5 min. After EGF stimulation, lysates were prepared and immunoprecipitation was carried out with anti-Myc antibody resin and immunoblotting with the indicated antibodies. Densitometric analysis was performed to determine the ratio of total ERK co-immunoprecipitation with ARL11 upon EGF treatment (*e*). Data represent mean ± S.D. (*error bars*) (*n* = 3) (**, *p* < 0.01; Student's *t* test). *f* and *g*, *ARL11*-Myc was cotransfected with HA-ERK2 (WT) or phospho-defective HA-ERK2 (T185A/Y187A) into HEK293T cells; lysates were immunoprecipitated with anti-HA antibody resin, and the precipitates were immunoblotted with the indicated antibodies. Densitometric analysis was performed to determine the ratio of ARL11 co-immunoprecipitated with WT or phospho-defective mutant of ERK2 (*g*). Data represent mean ± S.D. (*n* = 3) (**, *p* < 0.01; Student's *t* test). *h*, control or LPS-treated BMDMs were lysed, and the cell lysates were subjected to immunoprecipitation with anti-ARL11 antibody or anti-IgG control antibody, and the precipitates were immunoblotted with the indicated antibodies. *i*, HEK293T cells transfected with the indicated plasmids were either left untreated or treated with cyto D for 30 min. After cyto D treatment, lysates were prepared, and immunoprecipitation was carried out with anti-HA antibody resin and immunoblotting with the indicated antibodies. *j*, control, LPS-treated, and cyto D–treated BMDMs were lysed; the cell lysates were subjected to immunoprecipitation with anti-ARL11 antibody or anti-IgG control antibody; and the precipitates were immunoblotted with the indicated antibodies.

In our microscopy images, we also noted the presence of ARL11 beneath the plasma membrane, where it colocalized with the cortical actin filaments visualized in different cell types (Fig. 6*g*). Treatment of *ARL11*-transfected cells with cytochalasin D (cyto D) caused partial disruption of the actin cytoskeleton. The ARL11 pattern closely mirrored the drug-induced actin distribution, further supporting that ARL11 colocalizes with cortical actin (Fig. 6*h*). A similar distribution to the cortical actin structures has been reported previously for phospho-ERK1/2 (29–31). We found that epitope-tagged ERK2 was colocalized with ARL11 at the cortical actin structures (Fig. 6*i*). This led us to investigate whether there is a physical interaction between ARL11 and ERK. Among the MAPKs, ERK2 (or MAPK1), but not JNK1 or p38 kinase, was specifically co-immunoprecipitated with ARL11 (Fig. 7, *a* and *b*). Furthermore, using another ARL protein (ARL8b) as a control, we observed that ERK2 specifically co-immunoprecipitated ARL11 but not ARL8b (Fig. 7*c*).

Next, we investigated whether ARL11 interacts with the phosphorylated (active) form of ERK1/2. Interestingly, we found that transient stimulation of cells with EGF dramatically increased the total levels of endogenous ERK1/2 co-immunoprecipitated with ARL11. Further, using the anti-phospho-ERK1/2 antibody, we observed that this immunoprecipitated pool of ERK1/2 was phosphorylated (Fig. 7*d*; quantification shown in *e*). To corroborate these findings, we performed co-immunoprecipitation experiments in lysates of cells that had been pretreated with the MEK inhibitor U0126. As shown in Fig. S7*a*, interaction of ERK and ARL11 was abolished in cells pretreated with U0126, suggesting that ARL11 interacts with the phosphorylated form of ERK. We also verified that ERK activation is required for its interaction with ARL11 by using a phospho-defective mutant of ERK2 (T185A/Y187A) (Fig. S7*b*). Consistent with our earlier observations, ARL11 was co-immunoprecipitated with the WT but not with the phospho-defective mutant of ERK2 (T185A/Y187A), reinforcing that ARL11 specifically interacts with the activated form of ERK1/2 (Fig. 7*f*; quantification shown in *g*). In accordance with these results, we found that ERK1/2, but not p38 or JNK1/2, was co-immunoprecipitated with ARL11 in primary BMDMs (Fig. 7*h*, *middle* and *bottom panels*). Further, using the anti-phospho-ERK1/2 antibody, we observed that this immunoprecipitated pool of ERK1/2 was phosphorylated (Fig. 7*h*, *top panel*). Consistent with our findings that ARL11 interacts with ERK1/2 in its phosphorylated form, this interaction was significantly increased upon LPS stimulation in primary BMDMs (Fig. 7*h*). Because the ARL11-ERK interaction was detected within 10 min of LPS stimulation, this suggested formation of the complex near the plasma membrane. To investigate whether ERK1/2 and ARL11 interaction requires localization at the cortical actin structures, we pretreated cells with cyto D before immunoprecipitation for ERK1/2. Indeed, ARL11 and ERK interaction was abrogated upon depolymerization of actin filaments (Fig. 7*i*). These results were recapitulated under endogenous conditions in primary BMDMs, reinforcing the requirement of an intact actin cytoskeleton for ARL11 interaction with phospho-ERK (Fig. 7*j*). Taken together, our results suggest that ARL11 promotes or stabilizes LPS-dependent ERK1/2 phosphorylation, which is a prerequisite for the activation of pro-inflammatory immune responses in macrophages.

## Discussion

*ARLTS1*/*ARL11* was initially identified as a tumor suppressor at the gene locus *13q14.3*, a region frequently deleted in a variety of hematopoietic and solid tumors (3). The evidence that germ-line polymorphisms in the *ARL11* sequence were associated with familial risk for CLL and for breast, prostate, and colorectal cancers supported a causal role for *ARL11* in carcinogenesis (9–15). However, as most studies on this putative tumor suppressor involved ectopically expressing the protein in tumor cell lines, the endogenous function of ARL11 has remained uncharacterized so far. High sequence conservation of *ARL11* across evolution with orthologs present in diverse species, including fruit fly, zebrafish, mouse, and *Arabidopsis*, suggests an important cellular function of this gene (8).

Previous work had shown that *ARL11* transcripts are expressed in primary and secondary lymphoid organs and that *ARL11* expression is correlated with inflammatory pathways (8, 12). Our study provides the first evidence that ARL11 is endogenously expressed in macrophages and regulates the classic pro-inflammatory pathway of macrophage activation in response to pathogenic stimuli. Consequently, upon ARL11 depletion, expression of pro-inflammatory cytokines was severely reduced upon LPS treatment, and the phagocytic ability of macrophages was also severely impaired. Although phagocytosis was defective upon ARL11 depletion, survival of the phagocytosed bacteria was better in ARL11-depleted macrophages, due to impairment of antimicrobial effector responses, such as production of pro-inflammatory cytokines and antimicrobial compounds, including nitric oxide. Consistent with these observations, increased expression of ARL11 in macrophages was sufficient to induce their activation in the absence of pathogenic stimuli and expectedly led to a “macrophage exhaustion” phenotype, with no further increase in cytokine production observed upon LPS treatment. Our results hint at an as yet unknown regulatory mechanism that governs ARL11 function in macrophages in response to pathogenic stimuli. Mechanistically, we found that ARL11 promotes ERK1/2 phosphorylation in response to pathogenic stimuli, with *Arl11* overexpression circumventing the requirement for external stimuli to activate the ERK1/2 pathway. The latter observations suggest that ARL11 acts downstream of a TLR in the MAPK activation pathway. Consistent with this hypothesis, we did not observe a change in TLR4 surface levels in either ARL11-depleted or stably overexpressing cells.

To explore the mechanism of ARL11 action, we analyzed the subcellular localization of this protein that revealed its association with the actin-rich structures beneath the plasma membrane in addition to its nucleo-cytoplasmic localization. Importantly, ERK1/2 was colocalized with ARL11 on these cortical actin structures. Surprisingly, we found that phospho-ERK1/2 was present in a complex with ARL11, and this interaction probably occurs at the cortical actin filaments. Several lines of evidence suggest that this is the case: (*a*) interaction between ERK and ARL11 was enhanced upon EGF treatment; (*b*) interaction between the two proteins was disrupted upon treatment with the MEK inhibitor U0126 (which blocks ERK phosphorylation) and cyto D treatment (which disrupts the actin cytoskeleton); and (*c*) no interaction of the phospho-defective mutant of ERK (T185A/Y187A) with ARL11 was observed. Although detected in a complex, our yeast two-hybrid analysis suggests that ARL11 does not directly bind to ERK (data not shown).

ARL11 is a member of the Ras superfamily of small GTPases, which cycle between the GTP- and GDP-bound form and are functionally active in their GTP-bound state. Whether ARL11 associates with phospho-ERK in the GTP- or GDP-bound state or whether this binding is independent of the nucleotide status of ARL11 remains to be observed. This role of ARL11 was not specific to macrophages, as ectopic expression of *ARL11* in tumor cell lines was sufficient for ERK1/2 activation, an effect not observed upon transfection of other *ARL* family members. Our findings also explain the previously established role of ARL11 in promoting cancer cell apoptosis (3, 8, 32). It is known that duration, magnitude, and subcellular localization of activated ERK1/2 signal determine the cellular response. Transient ERK1/2 activation mediates survival and proliferation, whereas prolonged ERK1/2 activation induces the intrinsic and extrinsic apoptotic pathways in cancer cell lines (33). Thus, forced *ARL11* expression in tumor cells might promote sustained ERK1/2 activation, resulting in up-regulation of apoptotic markers. Indeed, in macrophages as well, we found that transfection of *Arl11* induced poly(ADP-ribose) polymerase and caspase-3 cleavage.

Our findings presented here give rise to several new questions. ARL11 interacts with ERK and promotes its phosphorylation. Is this due to direct stabilization of phospho-ERK1/2 levels by ARL11, or does ARL11 promote recruitment of scaffold proteins (such as IQGAP1) that enhance MAPK signaling? Does ARL11 also regulate alternative activation of macrophages observed upon stimulation with anti-inflammatory cytokines? What is the function of ARL11 in other immune cells, such as dendritic cells? Like other ARF family members, ARL11 also possesses the traditional sequence determinants that support the “ARF-specific” interswitch toggle mechanism for its activation. Thus, ARL11 GTP binding might require the presence of a guanine nucleotide exchange factor and conformational change of the amphipathic N-terminal helix induced upon recruitment to membranes. How the function of ARL11 is regulated in macrophages and the identity of the guanine nucleotide exchange factors and GTPase-activating proteins that regulate the GTP/GDP cycle of ARL11 remain to be identified. Further exploration of ARL11 function in immune cells will probably contribute answers to these questions.

## Experimental procedures

### Cell culture

For isolating and culturing BMDMs, bone marrow was harvested from tibiae and femurs of 6-weeks old C57BL/6 male mice. The cells inside the bones were flushed with growth medium RPMI 1640 (Lonza) supplemented with 10% heat-inactivated fetal bovine serum (FBS; Gibco), and red blood cells were lysed in ACK lysis buffer (155 mm NH_4_Cl, 10 mm KHCO_3_, 0.1 mm EDTA-2Na adjusted to pH 7.2) for 1 min. The remaining marrow cells were washed twice with 1× PBS and centrifuged at 2000 rpm for 5 min. Isolated bone marrow cells were seeded in 100-mm culture dishes in growth medium supplemented with 30 ng/ml recombinant murine macrophage colony-stimulating factor (34-8983; eBioscience) and cultured at 37 °C with 5% CO_2_ in a humidified cell culture chamber. After 3 days, half the volume of growth medium was replaced with fresh medium containing 60 ng/ml macrophage colony-stimulating factor, and the cells were allowed to grow until day 5. On day 5, BMDMs were trypsinized and seeded according to experimentation requirements.

RAW264.7, HeLa, HEK293T, MCF7, A549, and COS-7 cells were cultured in DMEM (Lonza) supplemented with 10% heat-inactivated FBS at 37 °C with 5% CO_2_ in a humidified cell culture chamber. For culturing THP-1 cells, RPMI 1640 (Lonza) supplemented with 10% heat-inactivated FBS was used. For differentiation of THP-1 monocytes to macrophages, 30 ng/ml PMA (Sigma) was added to the cell culture for 12 h, followed by a 12-h resting period. Each cell line was regularly screened for absence of mycoplasma contamination by using the MycoAlert mycoplasma detection kit (Lonza) and was passaged for no more than 15 passages. All of the cell lines were obtained from the American Type Culture Collection (ATCC).

### Antibodies and chemicals

The following antibodies were used in this study: mouse anti-HA (MMS-101P; Covance), rabbit anti-HA (H6908; Sigma-Aldrich), mouse anti-Myc (sc-40; Santa Cruz Biotechnology, Inc.), rabbit anti-CBP (07-482; Millipore), rabbit anti-*Salmonella* O-antigen (225341; BD Biosciences), rabbit anti-α-tubulin (ab15246; Abcam), mouse anti-GAPDH (sc-166574; Santa Cruz Biotechnology, Inc.), rabbit anti-histone H3 (9715; Cell Signaling Technology), rabbit anti-iNOS (sc-8310; Santa Cruz Biotechnology), mouse anti-CHOP (2895; Cell Signaling Technology), mouse anti-HO-1 (ADI-OSA-110-F; Enzo Life Sciences), rabbit anti-ARL11 (sc-83982; Santa Cruz Biotechnology), PE-conjugated rat anti-TLR4 (CD284) (145403; BioLegend), PE-conjugated rat anti-IgG1 (400508; BioLegend), rabbit anti-phospho-MEK1/2 (9154; Cell Signaling Technology), rabbit anti-MEK1/2 (9122; Cell Signaling Technology), rabbit anti-phospho-MKK3/MKK6 (9231; Cell Signaling Technology), rabbit anti-MKK3 (8535; Cell Signaling Technology), rabbit anti-phospho-p90RSK (11989; Cell Signaling Technology), rabbit anti-p90RSK (9355; Cell Signaling Technology), rabbit anti-phospho-NF-κB p65 (3033; Cell Signaling Technology), rabbit anti-NF-κB p65 (8242; Cell Signaling Technology), mouse anti-IκBα (4814; Cell Signaling Technology), MAPK family antibody kit (9926; Cell Signaling Technology), phospho-ERK1/2 pathway kit (9911; Cell Signaling Technology), phospho-MAPK family antibody kit (9910; Cell Signaling Technology), and apoptosis antibody kit (9915; Cell Signaling Technology). ARL11 (N-17) blocking peptide (sc-83982P) was purchased from Santa Cruz Biotechnology, Inc. All of the Alexa Fluor–conjugated secondary antibodies were purchased from Thermo Fisher Scientific. Horseradish peroxidase-conjugated goat anti-mouse and goat anti-rabbit antibodies were purchased from Jackson ImmunoResearch Laboratories. Alexa Fluor 568 phalloidin (A12380), hygromycin B (10687010), and DAPI (D1306) were purchased from Thermo Fisher Scientific. Staurosporine (S5921), cytochalasin D (cyto D, C2618), thapsigargin (T9033), U0126 monoethanolate (U120), PMA (P1585), LPS (L4391), Polybrene (H9268), and puromycin (P8833) were purchased from Sigma-Aldrich.

### Expression constructs

The human *ARL11* expression construct (RC203868) was obtained from Origene and cloned with a C-terminal HA or Myc tag into pcDNA3.1(−) vector (Invitrogen) using XhoI and BamHI restriction sites. To construct the C-terminal GFP-tagged human Arl11, the cloning was done in the pEGFP-N1 vector (Clontech) using XhoI and BamHI restriction sites. For performing the rescue experiments, the human *ARL11* gene with a C-terminal HA tag was cloned into the pCDH CMV MCS EF1-Hygro vector using NheI and BamHI restriction sites (System Biosciences). The mouse *Arl11* gene with a C-terminal HA or Myc tag was cloned into pcDNA3.1(−) using cDNA prepared from RAW264.7 macrophages. To create a C-terminal TAP-tagged mouse ARL11, the cloning was performed in the pCTAP-A vector (Agilent) using BamHI and XhoI restriction sites. HA-ERK2 (MAPK1/p42)-pcDNA3 and HA-JNK1-pcDNA3 were supplied by Dr. Steve Caplan (University of Nebraska Medical Center). HA-p38-pSG5 was kindly provided by Dr. Stephen Keyse (University of Dundee). All of the point mutants were designed and obtained using Stratagene site-directed mutagenesis kits (Agilent).

### Gene silencing and transient transfections

For siRNA-mediated gene silencing, siRNA oligonucleotides targeting human (L-018083-02) and mouse (L-055672-01) ARL11 transcripts were purchased from Dharmacon (GE Healthcare), and the knockdown was performed using DharmaFect 1 as per the manufacturer's instructions. For inhibiting the expression of CRM1, siRNA oligonucleotides (5′-GCTCAAGAAGTACTGACACAT-3′) custom synthesized from Dharmacon (GE Healthcare) were used. As a control, ON-TARGET-plus nontargeting pool (D-001810-10) was used. For stable knockdown, shRNA-mediated gene silencing was performed as described previously (34). The shRNA constructs were purchased from Sigma-Aldrich, and their target sequences were as follows: control shRNA, 5′-CAACAAGATGAAGAGCACCAA-3′; *Arl11* shRNA 1 (TRCN0000381486), 5′-TAATGATGGGCCTCGACTCTG-3′; and *Arl11* shRNA 2 (TRCN0000100452), 5′-GTACAAACTGAAAGGAAACTC-3′. For transient transfections, cells grown on tissue culture dishes or glass coverslips (VWR) were transfected with the desired constructs using X-tremeGENE HP DNA reagent (Roche Applied Science) for 14–16 h.

### Quantitative RT-PCR and cytokine determination by ELISA

Total RNA isolation from mammalian cells was performed using the RNeasy kit (74104; Qiagen) according to the manufacturer's directions. RNA was quantitated and used to prepare cDNA by using the SuperScript III First-Strand Synthesis System for RT-PCR (18080051; Invitrogen). The resulting cDNA was subjected to quantitative PCR using SYBR Green Universal Mix (11762100; Invitrogen) as per the manufacturer's instructions. Gene-specific primer sequences used for quantitative RT-PCR were as follows: human *ARL11*, 5′-GCAAGACCACGCTCCTTTACA-3′ (forward) and 5′-CTGTGCCTTCCAGATAGTCCTT-3′ (reverse); human CRM1, 5′-CTACATCTGCCTCTCCGTTGCT-3′ (forward) and 5′-CCAATACTTCCTCTGGTTTAGCC-3′ (reverse); human GAPDH, 5′-CATTTCCTGGTATGACAACGA-3′ (forward) and 5′-GTCTACATGGCAACTATGAG-3′ (reverse); mouse *Arl11*, 5′-CTCTTGAGGCTCCTGGACATGT-3′ (forward) and 5′-TCCAGCACGTACACAAGGAGGT-3′ (reverse); mouse TNFα, 5′-CTGGGACAGTGACCTGGACT-3′ (forward) and 5′-GCACCTCAGGGAAGAGTCTG-3′ (reverse); mouse IL-6, 5′-AGTTGCCTTCTTGGGACTGA-3′ (forward) and 5′-TCCACGATTTCCCAGAGAAC-3′ (reverse); mouse β-actin, 5′-GCTCTGGCTCCTAGCACCAT-3′ (forward) and 5′-GCCACCGATCCACACCGCGT-3′ (reverse).

Determination of IL-6 and TNFα level in culture supernatants of macrophages treated with LPS for different time periods or from *in vitro* infections was performed using mouse/human IL-6 or mouse/human TNFα BD OptEIA ELISA sets (BD Biosciences) according to the manufacturer's directions.

### Nitric oxide measurement

Control or ARL11-depleted macrophages were seeded in 12-well tissue culture dishes (0.15 × 10^6^ cells/well) in DMEM supplemented with 10% heat-inactivated FBS. After treatment of macrophages with LPS (1 μg/ml) or infection with *S. typhimurium* for the indicated time periods, the quantity of nitrite in the culture medium was measured as an indicator of nitric oxide production using Griess reagent (G4410; Sigma). Briefly, 100 μl of cell culture medium was mixed with 100 μl of Griess reagent. Subsequently, the mixture was incubated at room temperature for 5 min, and the absorbance at 540 nm was measured in a 96-well microplate reader (Tecan). Fresh culture medium was used as a blank in every experiment. The quantity of nitrite was determined from a sodium nitrite standard curve.

### Salmonella infection and gentamicin protection assay

To measure the intracellular *S. typhimurium* growth, the gentamicin protection assay was performed as described previously (35). Control, *Arl11*-silenced, and *Arl11* rescue macrophages were seeded in 24-well tissue culture dishes (0.15 × 10^6^ cells/well) in DMEM supplemented with 10% heat-inactivated FBS. Infection was performed using the stationary-phase *Salmonella* cultures (SL1344 strain kindly provided by Dr. John Brumell, Hospital for Sick Children, Toronto, Canada) incubated at 37 °C with shaking (OD = 1) and opsonized in PBS supplemented with 20% FBS for 20 min at 37 °C. After three washes in PBS, bacteria were resuspended in growth medium without antibiotics and added to the cells (multiplicity of infection of 50:1) and given a short centrifugation at 600 × *g* for 3 min at room temperature to synchronize the infection. The tissue culture dish was then incubated at 37 °C for 30 min to allow phagocytosis of bacteria. During the post-phagocytosis period, extracellular bacteria were removed by extensive washing using warm PBS, and to each well culture medium containing 50 μg/ml gentamicin was added for the next 90 min. After 2 h p.i., the concentration of gentamicin in the medium was decreased to 5 μg/ml. At the end of 20 h p.i. the cells were gently washed with PBS followed by lysis using PBS containing 0.1% Triton X-100 and 1% SDS for 5 min at room temperature. The resulting lysates were serially diluted and plated onto LB agar plates containing streptomycin. The plates were incubated at 37 °C for 16 h, and resultant colonies were counted to determine the number of cfu.

To check for the effect of MEK inhibitor (U0126) on *Salmonella* replication, macrophages were pretreated with either U0126 (10 μm) or DMSO (vehicle control) for 30 min and kept throughout the time until cells were lysed for dilution plating to determine cfu as described above.

### Phagocytosis assay

Control or ARL11-depleted RAW264.7 macrophages (3 × 10^5^ cells/well) were cultured in a 12-well plate tissue culture dish in DMEM supplemented with 10% heat-inactivated FBS at 37 °C with 5% CO_2_ in a humidified cell culture chamber. Following 24 h of LPS (1 μg/ml) treatment, culture dishes were incubated on ice for 10 min, and Alexa Fluor 488-conjugated *E. coli* (K-12 strain) BioParticles (E13231; Life Technologies) were added to each well at a multiplicity of infection of 10:1. To synchronize the update, the dishes were centrifuged at 300 × *g* for 5 min at 4 °C and further incubated on ice for 30 min to allow adherence of bioparticles to the cells. The dishes were then transferred to a cell culture chamber and incubated at 37 °C for 30 min to allow phagocytosis. After the incubation period, the dishes were washed three times in ice-cold PBS to remove excess bacteria before fluorescence measurement by flow cytometry (BD FACS Accuri; BD Biosciences).

### Flow cytometry

To assess expression of TLR4 on the surface of control, ARL11-depleted, or ARL11-overexpressing macrophages by flow cytometry, cells (0.5 × 10^6^) untreated or treated with 1 μg/ml LPS for 1 h were suspended in 100 μl of ice-cold FACS buffer (PBS + 10% FBS) containing a blocking agent (553141; BD Pharmingen) and placed on ice for 10 min. After incubation with blocking agent, cells were washed two times with ice-cold FACS buffer and further incubated on ice for 30 min in 100 μl of PE-conjugated rat anti-mouse TLR4 (CD284) or with isotype-specific PE-conjugated rat anti-mouse IgG1 prepared in FACS buffer. After washing three times with FACS buffer, cells were analyzed using a BD FACS Accuri cytometer (BD Biosciences).

### Cell lysates, co-immunoprecipitation, SDS-PAGE, and Western blotting

To prepare whole-cell protein lysates, cells after the indicated treatments were lysed in ice-cold radioimmune precipitation assay lysis buffer (10 mm Tris-Cl, pH 8.0, 1 mm EDTA, 0.5 mm EGTA, 1% Triton X-100, 0.1% SDS, 0.1% sodium deoxycholate, and 140 mm NaCl) supplemented with phosphatase inhibitor mixture (Roche Applied Science), protease inhibitor mixture (Sigma), 5 mm sodium orthovanadate, and 1 mm β-phosphoglycerate. After incubation on ice for 15 min, the lysed cells were centrifuged at 13,000 rpm for 15 min at 4 °C, and the supernatants were collected and quantitated (Bradford assay; Bio-Rad).

For co-immunoprecipitation analysis, primary BMDMs or HEK293T cells transfected with the indicated plasmids were lysed in TAP lysis buffer (20 mm Tris, pH 8.0, 150 mm NaCl, 0.5% Nonidet P-40, 1 mm MgCl_2_, 1 mm Na_3_VO_4_, 1 mm NaF, 1 mm phenylmethylsulfonyl fluoride, and protease inhibitor mixture; Sigma-Aldrich). To study the effect of inhibition of actin polymerization on protein–protein interactions, cells were treated with cyto D (5 μm) for 30 min before the addition of lysis buffer. The lysates were incubated with the indicated antibody-conjugated agarose beads at 4 °C with rotation for 3 h, followed by four washes in TAP wash buffer (20 mm Tris, pH 8.0, 150 mm NaCl, 0.1% Nonidet P-40, 1 mm MgCl_2_, 1 mm Na_3_VO_4_, 1 mm NaF, and 1 mm phenylmethylsulfonyl fluoride). The samples were then loaded on SDS-PAGE for further analysis.

SDS-PAGE and Western blotting were performed according to standard techniques. Briefly, protein samples separated on SDS-PAGE were transferred onto polyvinylidene difluoride membrane (Bio-Rad). Membranes were blocked overnight at 4 °C in blocking solution (10% skim milk prepared in PBS + 0.05% Tween 20). The indicated primary and secondary antibodies were prepared in PBS + 0.05% Tween 20. The membranes were washed for 10 min three times with PBS + 0.05% Tween 20 or PBS + 0.3% Tween 20 after a 2-h incubation with primary antibody and 1-h incubation with secondary antibody, respectively. The blots were developed using an acridan-based chemiluminescent horseradish peroxidase substrate (32132; Pierce) and X-ray films (Carestream).

### Subcellular fractionation

Nuclear/cytoplasmic separation was carried out by REAP (rapid, efficient, and practical) methodology as described previously (36). Briefly, cell pellets were resuspended in 900 μl of ice-cold 0.1% Nonidet P-40 (Sigma-Aldrich) in PBS and were triturated three times using 1000-μl microtips. One-third of each of the lysates (300 μl) was saved as “whole-cell lysate,” and the remaining lysate (600 μl) for each sample was subjected to centrifugation at 14,000 rpm for 10 s. After the spin, 300 μl of each supernatant was collected as “cytosolic fraction” and transferred to a fresh tube. The remaining supernatant was removed, and the pellet was washed once with 600 μl of 0.1% Nonidet P-40 in PBS. After centrifugation, the supernatant was removed, and the pellet was resuspended in 100 μl of 4× Laemmli buffer and labeled as “nuclear fraction.” To each “whole-cell lysate” and “cytosolic fraction” 100 μl of 4× Laemmli buffer was added. All of the fractions were sonicated for 5 s, followed by heating at 95 °C for 5 min, and then they were subjected to SDS-PAGE, followed by Western blotting.

### Immunofluorescence staining, confocal microscopy, and FRAP

Cells were fixed in 4% *p*-formaldehyde (PFA) in PHEM buffer (60 mm PIPES, 10 mm EGTA, 25 mm HEPES, and 2 mm MgCl_2_, final pH 6.8) for 10 min at room temperature. Post-fixation, cells were permeabilized for 5 min by adding permeabilization buffer (PBS + 0.2% Triton X-100). Following permeabilization, cells were washed three times in PBS and incubated in blocking solution (5% FBS in PBS) at room temperature for 30 min, followed by three washes with PBS. After this blocking step, cells were incubated with primary antibodies in staining solution (PBS + 0.05% Triton X-100 + 1% FBS) for 1 h at room temperature, washed three times with 1× PBS, and further incubated for 30 min with Alexa Fluor–conjugated secondary antibodies in staining solution. Cells were washed three times with 1× PBS and mounted in Fluoromount G (Southern Biotech). For actin staining, Alexa Fluor 568–conjugated Phalloidin (1:300 dilution) was added to the staining solution during the secondary antibody staining step. Single-plane confocal images were acquired using a Nikon A1R confocal microscope equipped with a Plan Apo VC ×60/1.4 numeric aperture oil immersion objective. For image acquisition, NIS-Elements AR 4.1 (Nikon) software was used. All images were captured to ensure that little or no pixel saturation is observed. The representative confocal images presented in the figures were imported into Adobe Photoshop CS and formatted to 300 dpi resolution.

For enumerating bacterial count by confocal microscopy, control, *Arl11*-silenced, and *Arl11* rescue macrophages were infected with GFP-expressing *Salmonella* using the protocol described above. After 1 and 6 h p.i., cells were fixed in 2.5% PFA in PBS at 37 °C for 20 min. Fixed cells were stained with Alexa Fluor 568–conjugated phalloidin to visualize the cell boundary, and the nucleus was stained using DAPI. To exclude extracellular bacteria from the count, coverslips were also stained for using anti-LPS antibody under nonpermeabilizing conditions. Using confocal microscopy, the intracellular bacteria were counted in ∼100 cells/experiment. These numbers were grouped according to the *key* in Fig. 4 and expressed as a percentage of the total infected cell population.

For determining the role of CRM1 in nuclear export of ARL11, HeLa cells expressing ARL11-GFP were transfected with control or CRM1 siRNA for 48 h. After siRNA treatment, cells were fixed in 4% PFA in PBS for 10 min, and the nuclei were stained with DAPI. The cells were imaged under a confocal microscope, and nuclear and cytoplasmic fluorescence intensities of ARL11-GFP were determined using ImageJ software (National Institutes of Health) and plotted as a ratio.

For FRAP experiments, HeLa cells were seeded into 35-mm live-cell imaging dishes (Eppendorf) and transiently transfected with the ARL11-GFP plasmid. Cells were visualized at 16 h post-transfection using a Nikon Eclipse Ti inverted microscope. Briefly, cells were monitored with a ×60/1.4 numerical aperture oil immersion objective, using the argon laser line at 488 nm, and a pre-bleach image at 2% laser intensity was acquired. Then photobleaching was performed by the 488-nm laser line at 100% intensity for 20 s at a region of interest (ROI) of ∼5 μm^2^ near either the center of the nucleus (for nuclear FRAP) or in the cytoplasm (for cytosolic FRAP) toward the cell periphery, followed by post-bleach image acquisition. Cells were closely monitored to rule out any artifact caused by photobleaching-induced toxicity and cell death. Without any time lapse post-bleach, fluorescence recovery was measured every 20 s for up to 10 min until the fluorescence recovery reached plateau stage. Images acquired were processed in ImageJ software, and the nuclear and total fluorescence intensities of ARL11-GFP were quantified at a given ROI at different time points during FRAP. Cytosolic intensity was calculated by subtracting nuclear intensity from total intensity. To plot for the change in nuclear and cytosolic fluorescence intensities during FRAP, initial pre-bleach intensity within the nucleus and cytosol were individually adjusted as 100%, and further intensities were calculated and plotted as a percentage of initial intensity remaining, thus giving a relative percentage fluorescence intensity.

### Ethics statement

This study was carried out in strict accordance with the guidelines issued by the Committee for the Purpose of Supervision of Experiments on Animals (1094 55/1999/CPCSEA) under the Prevention of Cruelty to Animals Act 1960 and amendments introduced in 1982 by the Ministry of Environment and Forest, Government of India. All protocols involving mouse experiments were approved by the institutional animal ethics committee of the Council of Scientific and Industrial Research-Institute of Microbial Technology (approval IAEC/16/12).

### Statistical analysis

Statistical analysis was carried out using GraphPad Prism version 6 software, with the two-tailed unpaired Student's *t* test to compare data from control cells *versus* treated cells at each corresponding time point. Data are shown as the mean ± S.D., with *p* values < 0.05 considered statistically significant.

## Author contributions

S. B. A. performed the majority of the experiments, analyzed the results, and prepared the figures. G. K., H. K., and A. K. conducted the protein–protein interaction experiments and provided critical molecular biology reagents. A. T. developed the concept, designed the experiments, and wrote the manuscript. All authors discussed the results and commented on the manuscript at all stages.

## Supplementary Material

Supporting Information

## Footnotes

7 Please note that the JBC is not responsible for the long-term archiving and maintenance of this site or any other third party hosted site.

## Footnotes

6 The abbreviations used are:

CLL: chronic lymphocytic leukemia
LPS: lipopolysaccharide
MAPK: mitogen-activated protein kinase
ERK: extracellular signal–regulated kinase
BMDM: bone marrow–derived mouse macrophage
TLR: Toll-like receptor
TNFα: tumor necrosis factor α
JNK: c-Jun N-terminal kinase
MAPKK: mitogen-activated protein kinase kinase
IL: interleukin
MEK: mitogen-activated protein kinase/extracellular signal-regulated kinase kinase
cfu: colony-forming units
p.i.: post-infection
HA: hemagglutinin
FRAP: fluorescence recovery after photobleaching
cyto D: cytochalasin D
EGF: epidermal growth factor
FBS: fetal bovine serum
DMEM: Dulbecco's modified Eagle's medium
PE: phycoerythrin
PFA: paraformaldehyde
DAPI: 4′,6-diamidino-2-phenylindole
ROI: region of interest
GAPDH: glyceraldehyde-3-phosphate dehydrogenase.

## References

[1] Donaldson J. G., and Jackson C. L. (2011) ARF family G proteins and their regulators: roles in membrane transport, development and disease. Nat. Rev. Mol. Cell Biol. 12, 362–375 10.1038/nrm3117 21587297 PMC3245550

[2] Khatter D., Sindhwani A., and Sharma M. (2015) Arf-like GTPase Arl8: moving from the periphery to the center of lysosomal biology. Cell Logist. 5, e1086501 10.1080/21592799.2015.1086501 27057420 PMC4820812

[3] Calin G. A., Trapasso F., Shimizu M., Dumitru C. D., Yendamuri S., Godwin A. K., Ferracin M., Bernardi G., Chatterjee D., Baldassarre G., Rattan S., Alder H., Mabuchi H., Shiraishi T., Hansen L. L., et al (2005) Familial cancer associated with a polymorphism in ARLTS1. N. Engl. J. Med. 352, 1667–1676 10.1056/NEJMoa042280 15843669

[4] La Starza R., Wlodarska I., Aventin A., Falzetti D., Crescenzi B., Martelli M. F., Van den Berghe H., and Mecucci C. (1998) Molecular delineation of 13q deletion boundaries in 20 patients with myeloid malignancies. Blood 91, 231–237 9414289

[5] Yin Z., Spitz M. R., Babaian R. J., Strom S. S., Troncoso P., and Kagan J. (1999) Limiting the location of a putative human prostate cancer tumor suppressor gene at chromosome 13q14.3. Oncogene 18, 7576–7583 10.1038/sj.onc.1203203 10602517

[6] Yendamuri S., Trapasso F., and Calin G. A. (2008) ARLTS1: a novel tumor suppressor gene. Cancer Lett. 264, 11–20 10.1016/j.canlet.2008.02.021 18375053

[7] Petrocca F., Iliopoulos D., Qin H. R., Nicoloso M. S., Yendamuri S., Wojcik S. E., Shimizu M., Di Leva G., Vecchione A., Trapasso F., Godwin A. K., Negrini M., Calin G. A., and Croce C. M. (2006) Alterations of the tumor suppressor gene ARLTS1 in ovarian cancer. Cancer Res. 66, 10287–10291 10.1158/0008-5472.CAN-06-2289 17079447

[8] Yendamuri S., Trapasso F., Ferracin M., Cesari R., Sevignani C., Shimizu M., Rattan S., Kuroki T., Dumon K. R., Bullrich F., Liu C. G., Negrini M., Williams N. N., Kaiser L. R., Croce C. M., and Calin G. A. (2007) Tumor suppressor functions of ARLTS1 in lung cancers. Cancer Res. 67, 7738–7745 10.1158/0008-5472.CAN-07-1481 17699778

[9] Frank B., Hemminki K., Meindl A., Wappenschmidt B., Klaes R., Schmutzler R. K., Untch M., Bugert P., Bartram C. R., and Burwinkel B. (2006) Association of the ARLTS1 Cys148Arg variant with familial breast cancer risk. Int. J. Cancer. 118, 2505–2508 10.1002/ijc.21687 16353159

[10] Frank B., Hemminki K., Brenner H., Hoffmeister M., Chang-Claude J., and Burwinkel B. (2006) ARLTS1 variants and risk of colorectal cancer. Cancer Lett. 244, 172–175 10.1016/j.canlet.2005.12.006 16488076

[11] Castellví-Bel S., Castells A., de Cid R., Muñoz J., Balaguer F., Gonzalo V., Ruiz-Ponte C., Andreu M., Llor X., Jover R., Bessa X., Xicola R. M., Pons E., Alenda C., Payá, et al (2007) Association of the ARLTS1 Cys148Arg variant with sporadic and familial colorectal cancer. Carcinogenesis 28, 1687–1691 10.1093/carcin/bgm098 17449901

[12] Siltanen S., Wahlfors T., Schindler M., Saramäki O. R., Mpindi J. P., Latonen L., Vessella R. L., Tammela T. L., Kallioniemi O., Visakorpi T., and Schleutker J. (2011) Contribution of ARLTS1 Cys148Arg (T442C) variant with prostate cancer risk and ARLTS1 function in prostate cancer cells. PLoS One 6, e26595 10.1371/journal.pone.0026595 22028916 PMC3197657

[13] Hamadou W. S., Besbes S., Mani R., Bourdon V., Ben Youssef Y., Achour B., Regaieg H., Eisinger F., Mari V., Gesta P., Dreyfus H., Bonadona V., Dugast C., Zattara H., Faivre L., et al (2017) ARLTS1, potential candidate gene in familial aggregation of hematological malignancies. Bull. Cancer 104, 123–127 10.1016/j.bulcan.2016.10.016 27866680

[14] Siltanen S., Fischer D., Rantapero T., Laitinen V., Mpindi J. P., Kallioniemi O., Wahlfors T., and Schleutker J. (2013) ARLTS1 and prostate cancer risk–analysis of expression and regulation. PLoS One 8, e72040 10.1371/journal.pone.0072040 23940804 PMC3734304

[15] Akisik E., Yazici H., and Dalay N. (2011) ARLTS1, MDM2 and RAD51 gene variations are associated with familial breast cancer. Mol. Biol. Rep. 38, 343–348 10.1007/s11033-010-0113-3 20358297

[16] Takeuchi O., and Akira S. (2010) Pattern recognition receptors and inflammation. Cell 140, 805–820 10.1016/j.cell.2010.01.022 20303872

[17] Endo M., Mori M., Akira S., and Gotoh T. (2006) C/EBP homologous protein (CHOP) is crucial for the induction of caspase-11 and the pathogenesis of lipopolysaccharide-induced inflammation. J. Immunol. 176, 6245–6253 10.4049/jimmunol.176.10.6245 16670335

[18] Endo M., Oyadomari S., Suga M., Mori M., and Gotoh T. (2005) The ER stress pathway involving CHOP is activated in the lungs of LPS-treated mice. J. Biochem. 138, 501–507 10.1093/jb/mvi143 16272146

[19] Yoo Y. H., Lim Y. J., Park S. E., Kim J. M., and Park Y. C. (2004) Overexpression of redox factor-1 negatively regulates NO synthesis and apoptosis in LPS-stimulated RAW 264.7 macrophages. FEBS Lett. 556, 39–42 10.1016/S0014-5793(03)01361-9 14706822

[20] Kavanagh E., Rodhe J., Burguillos M. A., Venero J. L., and Joseph B. (2014) Regulation of caspase-3 processing by cIAP2 controls the switch between pro-inflammatory activation and cell death in microglia. Cell Death Dis. 5, e1565 10.1038/cddis.2014.514 25501826 PMC4454160

[21] Conte D., Holcik M., Lefebvre C. A., Lacasse E., Picketts D. J., Wright K. E., and Korneluk R. G. (2006) Inhibitor of apoptosis protein cIAP2 is essential for lipopolysaccharide-induced macrophage survival. Mol. Cell. Biol. 26, 699–708 10.1128/MCB.26.2.699-708.2006 16382159 PMC1346893

[22] Lombardo E., Alvarez-Barrientos A., Maroto B., Boscá L., and Knaus U. G. (2007) TLR4-mediated survival of macrophages is MyD88 dependent and requires TNF-α autocrine signalling. J. Immunol. 178, 3731–3739 10.4049/jimmunol.178.6.3731 17339471

[23] Lloberas J., Valverde-Estrella L., Tur J., Vico T., and Celada A. (2016) Mitogen-activated protein kinases and mitogen kinase phosphatase 1: a critical interplay in macrophage biology. Front. Mol. Biosci. 3, 28 27446931 10.3389/fmolb.2016.00028 PMC4923182

[24] Weinstein S. L., Sanghera J. S., Lemke K., DeFranco A. L., and Pelech S. L. (1992) Bacterial lipopolysaccharide induces tyrosine phosphorylation and activation of mitogen-activated protein kinases in macrophages. J. Biol. Chem. 267, 14955–14962 1321821

[25] Pomerantz R. J., Feinberg M. B., Trono D., and Baltimore D. (1990) Lipopolysaccharide is a potent monocyte/macrophage-specific stimulator of human immunodeficiency virus type 1 expression. J. Exp. Med. 172, 253–261 10.1084/jem.172.1.253 2193097 PMC2188186

[26] Jones E., Adcock I. M., Ahmed B. Y., and Punchard N. A. (2007) Modulation of LPS stimulated NF-κB mediated nitric oxide production by PKCϵ and JAK2 in RAW macrophages. J. Inflamm. (Lond.) 4, 23 10.1186/1476-9255-4-23 18036230 PMC2211292

[27] Xiao Y. Q., Malcolm K., Worthen G. S., Gardai S., Schiemann W. P., Fadok V. A., Bratton D. L., and Henson P. M. (2002) Cross-talk between ERK and p38 MAPK mediates selective suppression of pro-inflammatory cytokines by transforming growth factor-β. J. Biol. Chem. 277, 14884–14893 10.1074/jbc.M111718200 11842088

[28] Baeuerle P. A., and Baltimore D. (1996) NF-κB: ten years after. Cell 87, 13–20 10.1016/S0092-8674(00)81318-5 8858144

[29] McKay M. M., and Morrison D. K. (2007) Integrating signals from RTKs to ERK/MAPK. Oncogene 26, 3113–3121 10.1038/sj.onc.1210394 17496910

[30] Mendoza M. C., Er E. E., Zhang W., Ballif B. A., Elliott H. L., Danuser G., and Blenis J. (2011) ERK-MAPK drives lamellipodia protrusion by activating the WAVE2 regulatory complex. Mol. Cell 41, 661–671 10.1016/j.molcel.2011.02.031 21419341 PMC3078620

[31] Kang H., Zhang J., Wang B., Liu M., Zhao J., Yang M., and Li Y. (2017) Puerarin inhibits M2 polarization and metastasis of tumor-associated macrophages from NSCLC xenograft model via inactivating MEK/ERK 1/2 pathway. Int. J. Oncol. 50, 545–554 10.3892/ijo.2017.3841 28101568

[32] Yang X. Y., Yu H., Lin W., and Peng Z. L. (2009) [Effects of ARLTS1 gene on growth and apoptosis of epithelial ovarian cancer SKOV3 cells]. Sichuan Da Xue Xue Bao Yi Xue Ban 40, 6–10 19292033

[33] Cagnol S., and Chambard J. C. (2010) ERK and cell death: mechanisms of ERK-induced cell death: apoptosis, autophagy and senescence. FEBS J. 277, 2–21 10.1111/j.1742-4658.2009.07366.x 19843174

[34] Marwaha R., Arya S. B., Jagga D., Kaur H., Tuli A., and Sharma M. (2017) The Rab7 effector PLEKHM1 binds Arl8b to promote cargo traffic to lysosomes. J. Cell Biol. 216, 1051–1070 10.1083/jcb.201607085 28325809 PMC5379943

[35] Sindhwani A., Arya S. B., Kaur H., Jagga D., Tuli A., and Sharma M. (2017) *Salmonella* exploits the host endolysosomal tethering factor HOPS complex to promote its intravacuolar replication. PLoS Pathog. 13, e1006700 10.1371/journal.ppat.1006700 29084291 PMC5679646

[36] Suzuki K., Bose P., Leong-Quong R. Y., Fujita D. J., and Riabowol K. (2010) REAP: A two minute cell fractionation method. BMC Res. Notes 3, 294 10.1186/1756-0500-3-294 21067583 PMC2993727

[37] Heng T. S., Painter M. W., and Immunological Genome Project Consortium (2008) The Immunological Genome Project: Networks of gene expression in immune cells. Nat. Immunol. 9, 1091–1094 10.1038/ni1008-1091 18800157

