# Supplementary material for: ARL11 regulates lipopolysaccharide-stimulated macrophage activation by promoting mitogen-activated protein kinase (MAPK) signaling
Source: J Biol Chem. 2018 Apr 4;293(25):9892–909. doi: 10.1074/jbc.RA117.000727 (PMC6016484; doi:10.1074/jbc.RA117.000727)
Supplement: Supporting Information [file supp_293_25_9892__index.html]

Arl11 regulates lipopolysacchride-stimulated macrophage activation by promoting MAPK signaling — Arl11 regulates ERK signaling in macrophages — ARL11 regulates lipopolysaccharide-stimulated macrophage activation by promoting mitogen-activated protein kinase (MAPK) signaling — ARL11 regulates ERK signaling in macrophages — Supporting Information 

# ARL11 regulates lipopolysaccharide-stimulated macrophage activation by promoting mitogen-activated protein kinase (MAPK) signaling

## Supporting Information

- Video S1 - Nuclear FRAP analysis of a HeLa cell transiently expressing Arl11-GFP.
- Video S2 - Cytosolic FRAP analysis of a HeLa cell transiently expressing Arl11-GFP.
- Fig. S1 - Arl11 expression is increased upon LPS treatment but not by ER or oxidative stress activation in macrophages.
- Fig. S2 - Arl11 depletion does not affect cell proliferation or surface TLR4 levels in RAW264.7 macrophages.
- Fig. S3 - Arl11 silencing impairs effector functions of PMA-differentiated THP-1 cells.
- Fig. S4 - Arl11 depletion does not impair MEK1/2 and MKK3/6 phosphorylation in LPS stimulated macrophages.
- Fig. S5 - ERK signaling regulates intracellular Salmonella replication in macrophages.
- Fig. S6 - Arl11 actively shuttles in and out of the nucleus and requires CRM1 activity for nuclear export.
- Fig. S7 - Arl11 specifically interacts with the phosphorylated form of ERK.
