# Supplementary material for: ARL11 regulates lipopolysaccharide-stimulated macrophage activation by promoting mitogen-activated protein kinase (MAPK) signaling
Source: J Biol Chem. 2018 Apr 4;293(25):9892–909. doi: 10.1074/jbc.RA117.000727 (PMC6016484; doi:10.1074/jbc.RA117.000727)
Supplement: Supporting Information [file supp_RA117.000727_133585_1_supp_100253_p5wtp8.pdf]

SUPPORTING INFORMATION

Arl11 regulates lipopolysacchride-stimulated macrophage activation by promoting MAPK signaling

Subhash B. Arya, Gaurav Kumar, Harmeet Kaur, Amandeep Kaur, and Amit Tuli

Fig. S1

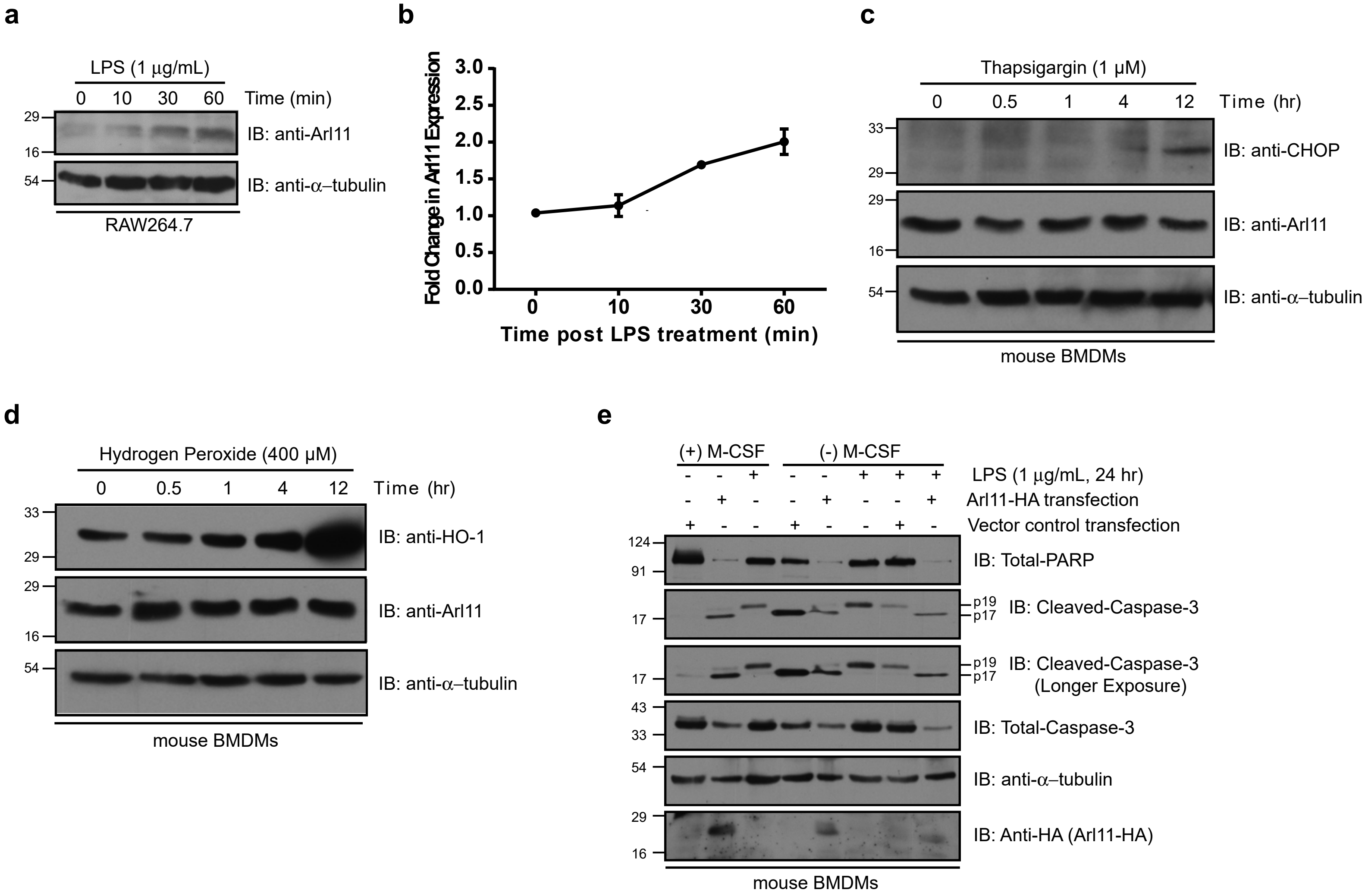

Fig. S1: Arl11 expression is increased upon LPS treatment but not by ER or oxidative stress activation in macrophages. a and **b)** RAW264.7 cells were treated with LPS for different time periods as indicated and Western blot analysis for Arl11 expression was performed. Densitometric analysis of fold change in Arl11 protein levels upon LPS stimulation in RAW264.7 cells compared with untreated controls is plotted (**b**). Western blot for α-tubulin was performed to show equal amount of protein loading. **c** and **d)** Primary BMDMs were treated with thapsigargin (1 µM) or hydrogen peroxide (400 µM) for the indicated periods, then cell lysates were subjected to Western blot analysis using antibodies against Arl11, CHOP (positive control for ER stress induction upon thapsigargin treatment), HO-1 (positive control for induction of oxidative stress upon hydrogen peroxide treatment) and α-tubulin (equal protein loading control). **e)** Overexpression of Arl11 induces apoptosis in macrophages. Primary BMDMs transfected with empty vector (control) or mArl11-HA expressing plasmid were incubated in media with (*lanes* 1-3) or without M-CSF (*lanes* 4-8). The cells were either left untreated or treated with LPS (100 ng/mL) for 24 hours as indicated. Then cell lysates were prepared and analyzed for induction of apoptosis by checking the levels of total-PARP, total-caspase-3 and cleaved-caspase-3 by Western blot analysis. As shown in *lane* 2, overexpression of Arl11 induced apoptosis in BMDMs as evident by reduced levels of total-PARP and increased caspase-3 cleavage. Of note, upon Arl11 transfection in BMDMs, processing of the p19 caspase-3 fragment to the active p17 fragment was enhanced (compare *lanes* 1 and 2). In agreement with previously reported LPS-mediated survival effect in BMDMs, cleavage of PARP and caspase-3 (p19 conversion to p17) was low (*lane* 3). Also, overexpressed Arl11 was able to induce apoptosis even after LPS stimulation of macrophages upon M-CSF withdrawal (compare *lanes* 7 and 8).
