# Supplementary material for: ARL11 regulates lipopolysaccharide-stimulated macrophage activation by promoting mitogen-activated protein kinase (MAPK) signaling
Source: J Biol Chem. 2018 Apr 4;293(25):9892–909. doi: 10.1074/jbc.RA117.000727 (PMC6016484; doi:10.1074/jbc.RA117.000727)
Supplement: Supporting Information [file supp_RA117.000727_133585_1_supp_100254_p5wtp8.pdf]

Fig. S2

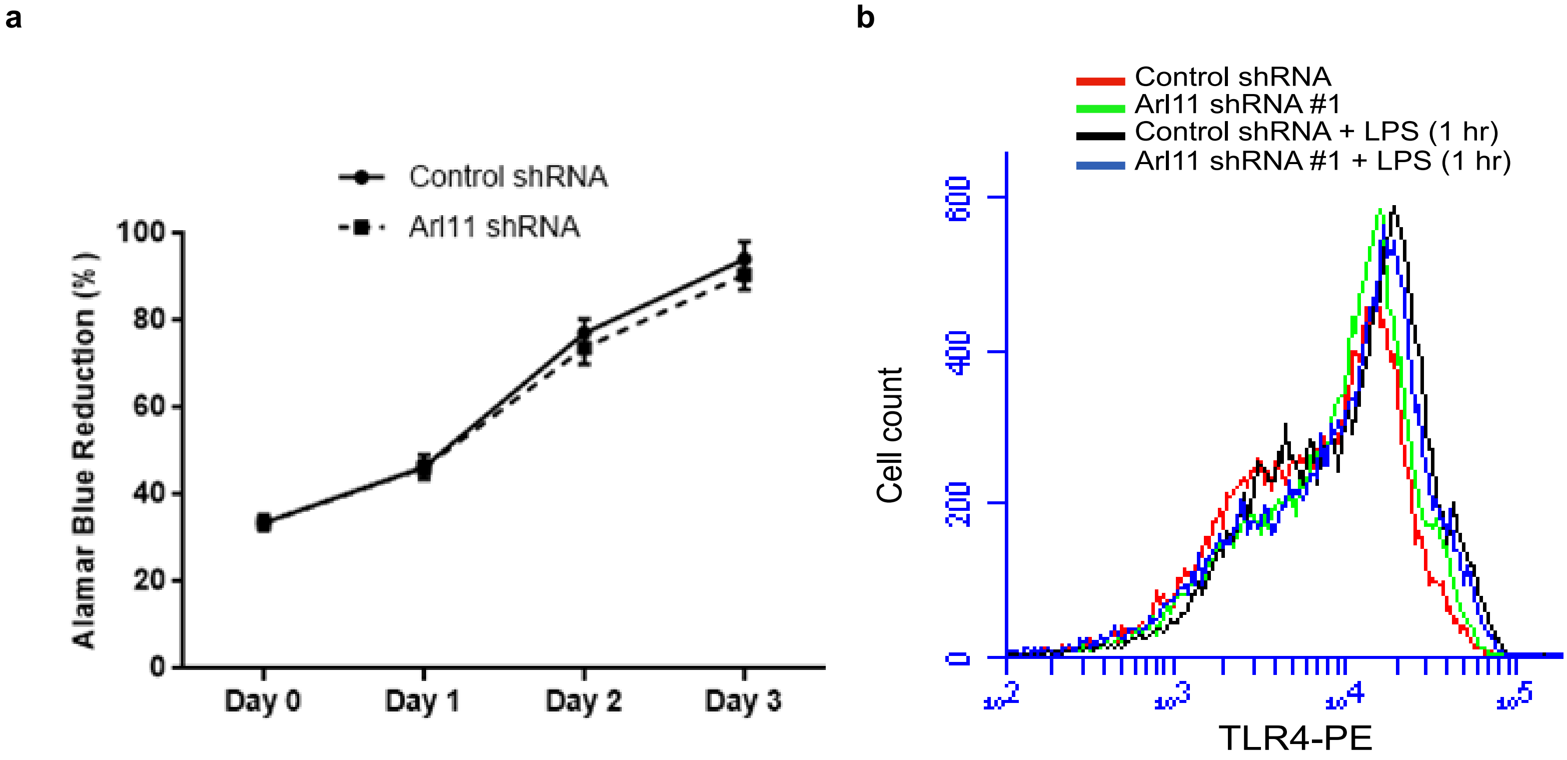

Fig. S2: Arl11 depletion does not affect cell proliferation or surface TLR4 levels in RAW264.7 macrophages. a) Arl11 silencing does not affect cell proliferation rate of macrophages. Control shRNA- and Arl11 shRNA-transfected RAW264.7 cells were seeded in 96-well plates and cell viability assessment was performed using the Alamar blue assay. **b)** Arl11 silencing does not affect TLR4 cell surface expression. Control shRNA- and Arl11 shRNA-transfected RAW264.7 cells untreated or stimulated with LPS for 1 hour and TLR4 surface expression was analyzed by flow cytometry.
