# Supplementary material for: ARL11 regulates lipopolysaccharide-stimulated macrophage activation by promoting mitogen-activated protein kinase (MAPK) signaling
Source: J Biol Chem. 2018 Apr 4;293(25):9892–909. doi: 10.1074/jbc.RA117.000727 (PMC6016484; doi:10.1074/jbc.RA117.000727)
Supplement: Supporting Information [file supp_RA117.000727_133585_1_supp_100255_p5wtp8.pdf]

Fig. S3

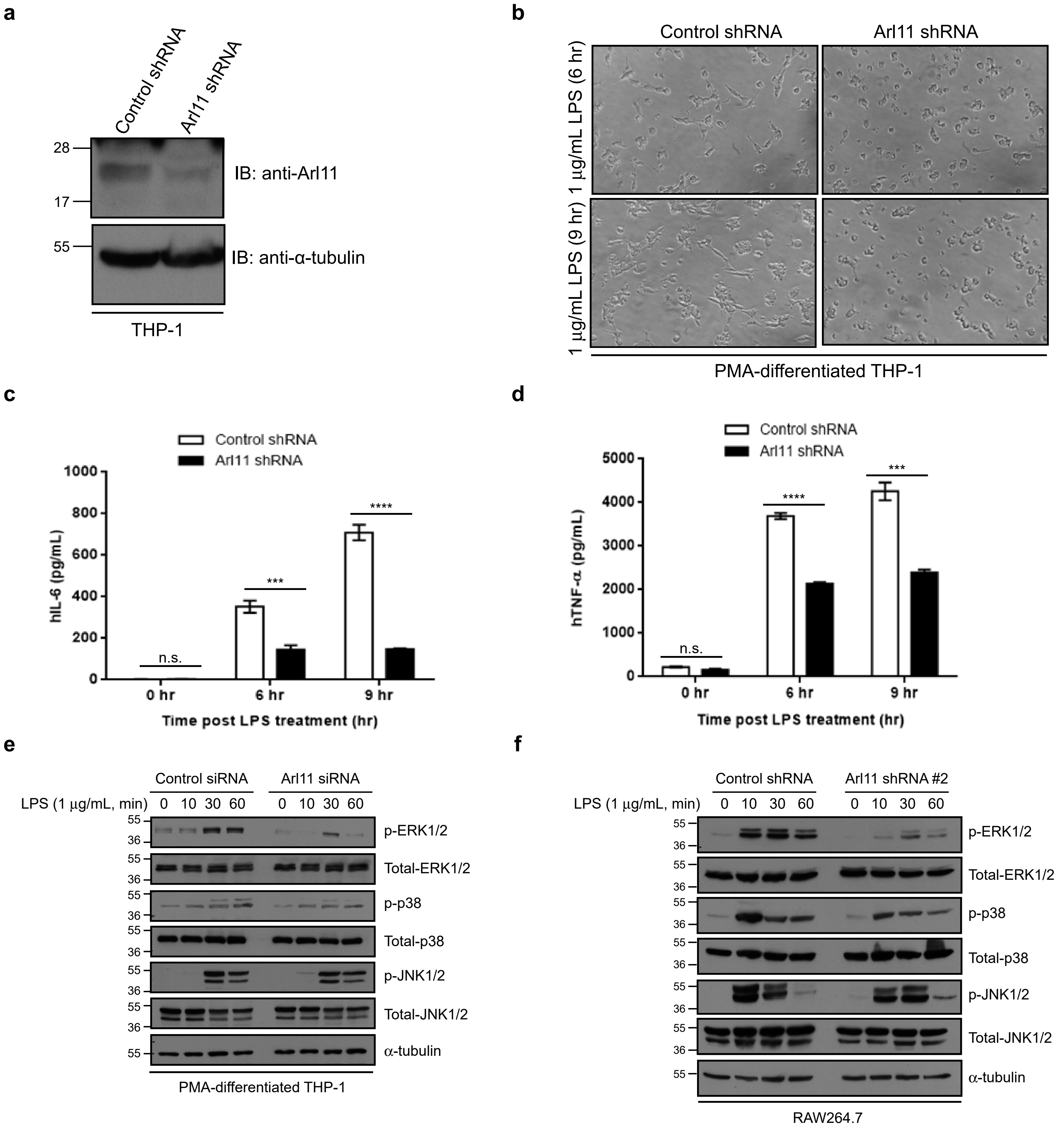

Fig. S3: Arl11 silencing impairs effector functions of PMA-differentiated THP-1 cells. a) PMA-differentiated THP-1 cells were transfected with control shRNA or Arl11 shRNA, and the lysates were IB with anti-Arl11 antibody for assessing the knockdown efficiency and anti-α-tubulin antibody for equal protein loading. **b)** Phase-contrast micrographs of control shRNA- and Arl11 shRNA-transfected THP-1 cells stimulated with LPS. **c** and **d)** Control and Arl11-silenced THP-1 macrophages were stimulated with 1 μg/mL LPS for the indicated time periods and supernatants from the cultures were collected and the concentration of IL-6 and TNF-α was measured by ELISA. Data shown represents mean ± SD (n=3; n.s., not significant; ***P < 0.001; ****P < 0.0001; Student's *t* test). **e)** THP-1 macrophages were transfected with either control siRNA or Arl11-specific siRNA. Post 72 hours, cells were stimulated with LPS for the indicated time periods, and the lysates were prepared and IB with indicated antibodies. **f)** Control shRNA- and Arl11 shRNA #2-transfected RAW264.7 cells were treated with LPS for different time periods, and lysates were prepared and IB with indicated antibodies.
