# Supplementary material for: ARL11 regulates lipopolysaccharide-stimulated macrophage activation by promoting mitogen-activated protein kinase (MAPK) signaling
Source: J Biol Chem. 2018 Apr 4;293(25):9892–909. doi: 10.1074/jbc.RA117.000727 (PMC6016484; doi:10.1074/jbc.RA117.000727)
Supplement: Supporting Information [file supp_RA117.000727_133585_1_supp_100256_p5wtp8.pdf]

Fig. S4

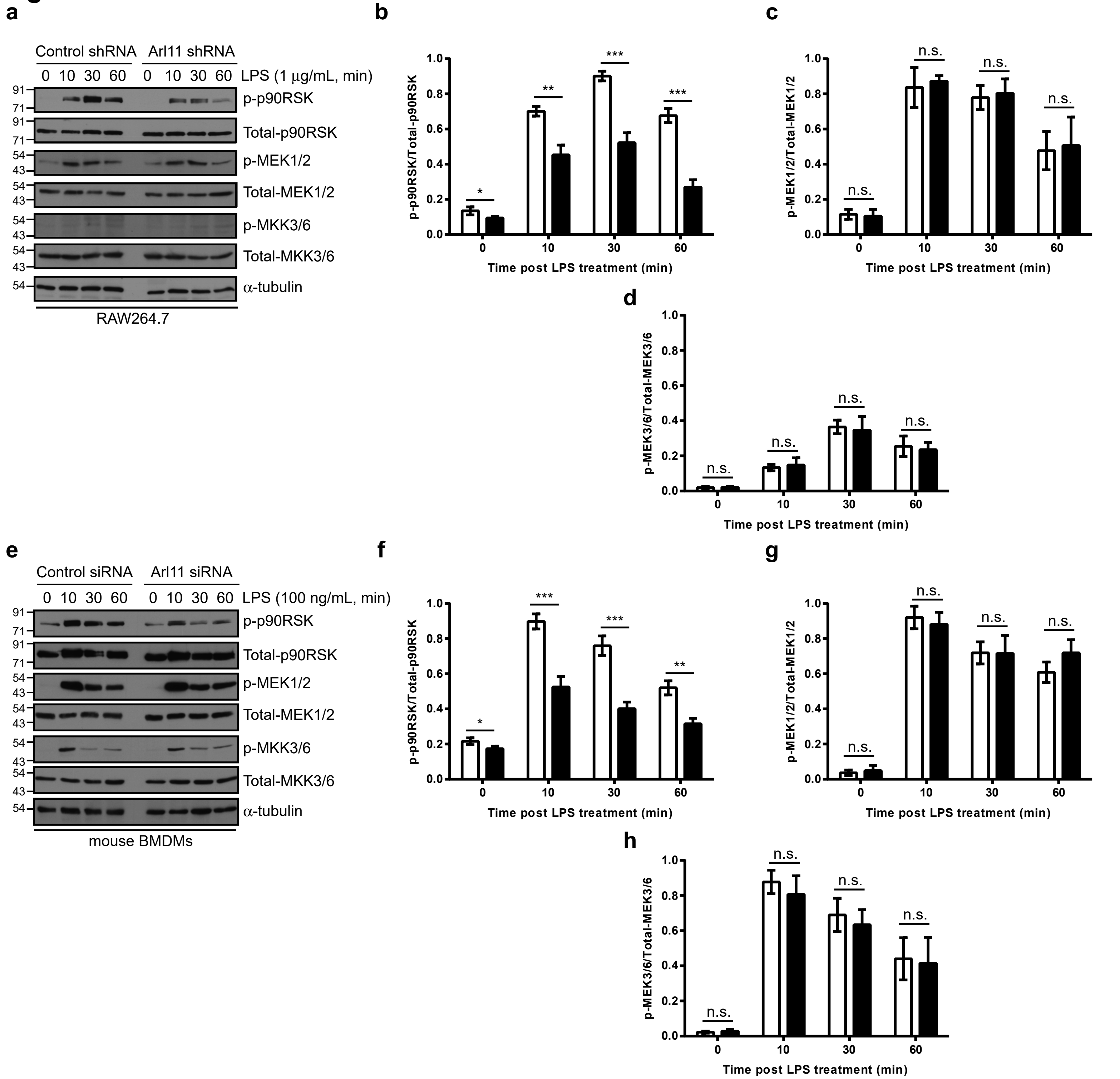

Fig. S4: Arl11 depletion does not impair MEK1/2 and MKK3/6 phosphorylation in LPS stimulated macrophages. a) Control shRNA- and Arl11 shRNA-transfected RAW264.7 cells were treated with LPS for different time periods, and lysates were prepared and blotted with indicated antibodies. **b-d)** Densitometric analysis was performed to determine the ratio of p-p90RSK to total-p90RSK (**b**), p-MEK1/2 to total-MEK1/2 (**c**), and p-MKK3/6 to total-MKK3/6 (**d**) in control shRNA- and Arl11 shRNA-transfected RAW264.7 cells treated with LPS for different time periods as indicated. **e)** Primary BMDMs were transfected with control- or Arl11-siRNA. Post 72 hours of siRNA transfections, cells were stimulated with LPS for the indicated time periods, and the lysates were prepared and blotted with indicated antibodies. **f-h)** Densitometric analysis was performed to determine the ratio of p-p90RSK to total-p90RSK (**f**), p-MEK1/2 to total-MEK1/2 (**g**), and p-MKK3/6 to total-MKK3/6 (**h**) in control- and Arl11-siRNA transfected BMDMs cells treated with 100 ng/mL LPS for different time periods as indicated. Note: Phosphorylation of p90RSK, a downstream substrate of ERK1/2, was inhibited in Arl11 silenced-BMDMs and -RAW264.7 cells upon LPS treatment. Data shown represents mean ± SD (n=3; n.s., not significant; *P<0.05; **P<0.01; ***P < 0.001; Student's *t* test).
