# Supplementary material for: ARL11 regulates lipopolysaccharide-stimulated macrophage activation by promoting mitogen-activated protein kinase (MAPK) signaling
Source: J Biol Chem. 2018 Apr 4;293(25):9892–909. doi: 10.1074/jbc.RA117.000727 (PMC6016484; doi:10.1074/jbc.RA117.000727)
Supplement: Supporting Information [file supp_RA117.000727_133585_1_supp_100257_p5wtp8.pdf]

Fig. S5

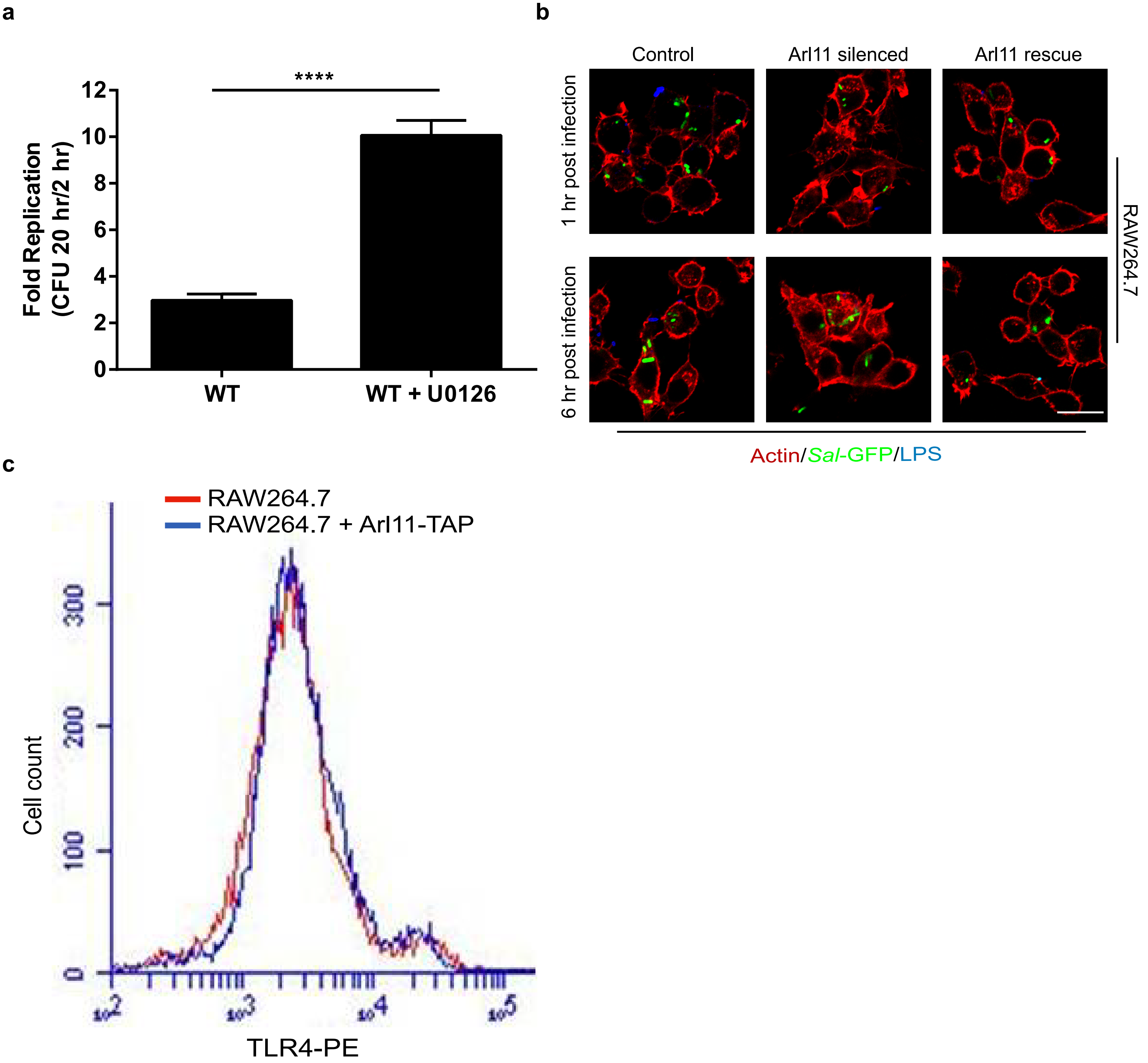

Fig. S5: ERK signaling regulates intracellular *Salmonella* replication in macrophages. a) Wild type RAW264.7 cells treated with DMSO (vehicle control) or with U0126 inhibitor (10 μM) were infected with *Salmonella* and the fold change in recoverable colony forming units (CFU) was calculated (20 hr/2 hr p.i.) by Gentamicin protection assay. Data shown represents mean ± SD (n=3; ****P < 0.0001; Student's *t* test). **b)** Representative confocal micrographs of control-, Arl11 silenced- and Arl11 rescued-RAW264.7 cells infected with GFP-expressing *Salmonella* (green). At different times after infection, cells were fixed and stained with anti-*Salmonella* antibody (blue) to mark extracellular or non-internalized bacteria, and phalloidin (red actin staining) to mark cell boundary. Bar, 10 μm. **c)** Arl11 overexpression does not affect TLR4 cell surface expression. Surface expression of TLR4 in vector- and Arl11-TAP-transfected RAW264.7 cells was analyzed by flow cytometry.
