# Supplementary material for: ARL11 regulates lipopolysaccharide-stimulated macrophage activation by promoting mitogen-activated protein kinase (MAPK) signaling
Source: J Biol Chem. 2018 Apr 4;293(25):9892–909. doi: 10.1074/jbc.RA117.000727 (PMC6016484; doi:10.1074/jbc.RA117.000727)
Supplement: Supporting Information [file supp_RA117.000727_133585_1_supp_100258_p5wtp8.pdf]

Fig. S6

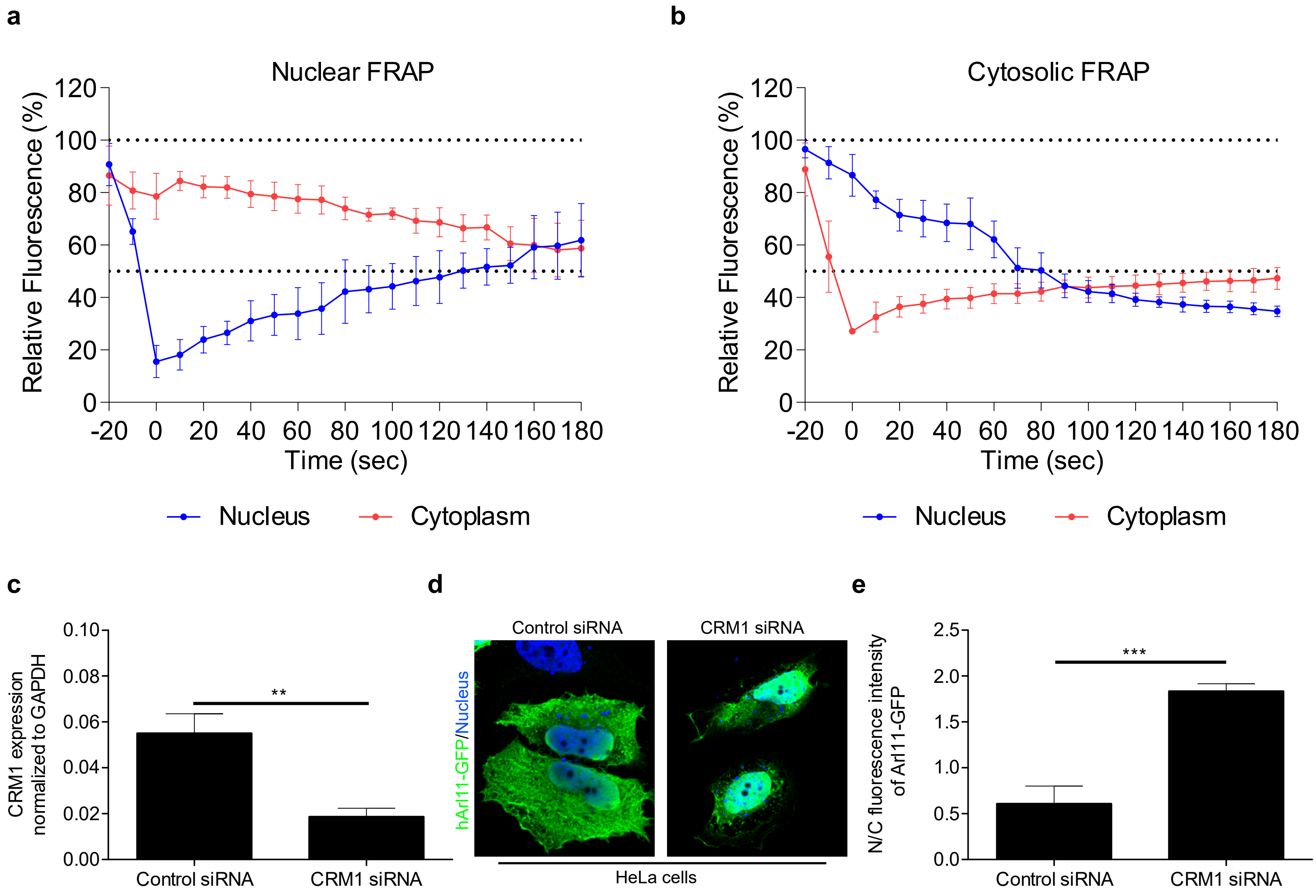

Fig. S6: Arl11 actively shuttles in and out of the nucleus and requires CRM1 activity for nuclear export. a and **b)** Quantification of nuclear and cytoplasmic FRAP analysis on HeLa cells transfected with Arl11-GFP plasmid. The nuclear and total fluorescence intensities of Arl11-GFP was quantified at a given ROI at different time points during FRAP using ImageJ software. Cytosolic intensity was calculated by subtracting nuclear intensity from total intensity. To plot for the change in nuclear and cytosolic fluorescence intensities, initial pre-bleach intensity within nucleus and cytosol were individually adjusted to 100% and further intensities were calculated and plotted as percentage of initial intensity remaining. **c-e)** Silencing of CRM1 expression prevents nuclear export of Arl11. Silencing of CRM1 expression in HeLa cells was confirmed by qRT-PCR (**c**). HeLa cells expressing Arl11-GFP were transfected with control- or CRM1-siRNA for 48 hours. Post siRNA treatment, cells were fixed in 4% PFA in PBS for 10 minutes and the nuclei were stained with DAPI (**d**). The cells were imaged under confocal microscope, and nuclear and cytoplasmic fluorescence intensities of Arl11-GFP were determined using ImageJ software (NIH) and plotted as ratio (**e**). Data shown represents mean ± SD (n=3; **P<0.01; ***P < 0.001; Student's *t* test).
