# Supplementary material for: ARL11 regulates lipopolysaccharide-stimulated macrophage activation by promoting mitogen-activated protein kinase (MAPK) signaling
Source: J Biol Chem. 2018 Apr 4;293(25):9892–909. doi: 10.1074/jbc.RA117.000727 (PMC6016484; doi:10.1074/jbc.RA117.000727)
Supplement: Supporting Information [file supp_RA117.000727_133585_1_supp_100259_p5wtp8.pdf]

Fig. S7

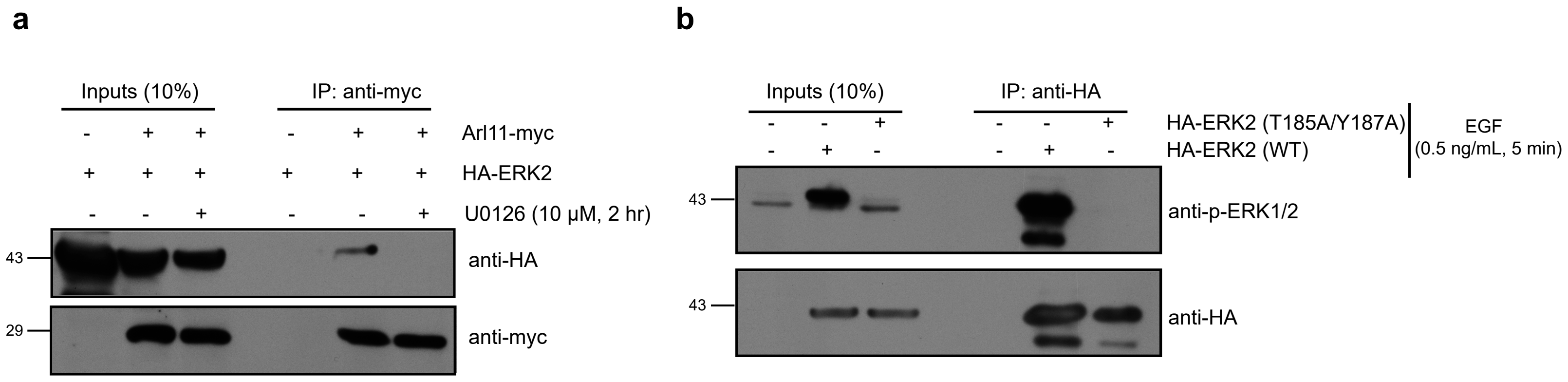

Fig. S7: Arl11 specifically interacts with the phosphorylated form of ERK. a) Arl11-myc was co-transfected with HA-ERK2 into HEK293T cells. After 12 hours, cells were left untreated or treated with MEK1/2 inhibitor (U0126). After 2 hours, cells were lysed and lysates were subjected to IP with anti-myc antibody resin and the precipitates were IB with the indicated antibodies. **b)** HEK293T cells transfected with HA-ERK2 (WT) or phospho-defective HA-ERK2 (T185A/Y187A) were treated with EGF (0.5 ng/mL) for 5 minutes. Post EGF treatment, lysates were prepared and IP with anti-HA antibody resin, and the precipitates were IB with the indicated antibodies.

SUPPORTING ONLINE VIDEOS

Video S1: Nuclear FRAP analysis of a HeLa cell transiently expressing Arl11-GFP. Nucleus was photobleached to background levels and recovery was monitored every 20 seconds for upto 10 minutes until the fluorescence recovery reaches plateau stage. A total of 32 frames were captured and the movie is shown at 6.4 frames per second.

Video S2: Cytosolic FRAP analysis of a HeLa cell transiently expressing Arl11-GFP. A region within the cytosol was photobleached to background levels and recovery was monitored every 20 seconds for upto 10 minutes until the fluorescence recovery reaches plateau stage. A total of 32 frames were captured and the movie is shown at 6.4 frames per second.
